# Role of lipocalin-2 in amyotrophic lateral sclerosis

**DOI:** 10.3389/fnagi.2025.1672903

**Published:** 2025-10-02

**Authors:** Zhuoya Wang, Wen Cao, Dongsheng Fan

**Affiliations:** ^1^Department of Neurology, Peking University Third Hospital, Beijing, China; ^2^Beijing Key Laboratory of Biomarker and Translational Research in Neurodegenerative Diseases, Beijing, China; ^3^Key Laboratory for Neuroscience, National Health Commission/Ministry of Education, Peking University, Beijing, China

**Keywords:** amyotrophic lateral sclerosis, lipocalin-2, neutrophil gelatinase-associated lipocalin, neuroinflammation, iron metabolism, cell death, peripheral immunity

## Abstract

Amyotrophic lateral sclerosis (ALS) is a progressive neurodegenerative disease characterized pathologically by degeneration of upper and lower motor neurons, ultimately leading to muscle weakness and respiratory failure. Lipocalin-2 (LCN2) is a secreted protein involved in lipid transport that plays a key role in inflammatory responses and the regulation of iron homeostasis. The role of LCN2 in ALS has attracted increasing attention, as significantly elevated LCN2 expression has been observed in the blood and postmortem tissues of ALS patients. Functionally, LCN2 participates in neuroinflammation, iron dysregulation, cell death, and peripheral immune immunity, proposing a central-peripheral linkage hypothesis mediated by LCN2. Clinically, LCN2 shows promise as both a biomarker and a therapeutic target, with multiple strategies demonstrating potential to mitigate ALS pathology. Moving forward, it is essential to integrate multi-omics to deeply decipher LCN2-mediated molecular networks, advance patient stratification, and accelerate its clinical translation.

## 1 Introduction

Amyotrophic lateral sclerosis (ALS) is a fatal neurodegenerative disease and the most common form of motor neuron disease. It is typically characterized by progressive degeneration of both upper and lower motor neurons, leading to muscle weakness and ultimately death due to respiratory failure (Feldman et al., 2022). Globally, the annual incidence of ALS is approximately 1.68 per 100,000 individuals (Marin et al., 2017). The risk of developing ALS increases with age, peaking between 60 and 79 years (Marin et al., 2018; Mehta et al., 2018). Based on the presence or absence of a family history, ALS can be classified as familial (about 10%) or sporadic (about 90%). Familial ALS is often associated with inherited mutations in specific genes (Cady et al., 2015; Chia et al., 2018; Goutman et al., 2018). At present, there is no cure for ALS. Existing treatments focus on slowing disease progression and improving patients’ quality of life (Klavžar et al., 2020).

It is worth noting that the clinical manifestations of ALS are highly heterogeneous, mainly in terms of age of onset, site of onset, rate of progression and survival (Ilieva et al., 2023). Recent studies suggest that immune system dysfunction may explain part of this heterogeneity (Nardo et al., 2013). Although ALS is not considered a classic autoimmune disease, activation of both central and peripheral immune responses can accelerate motor neuron injury via inflammatory mechanisms and contribute to disease progression (Beers and Appel, 2019). It has been found that ALS patients have significant central nervous system (CNS) neuroinflammation (Liu et al., 2021), as well as dysregulation of immune cells and inflammatory factors in the peripheral blood (Murdock et al., 2017). However, current studies on the role of immunity in ALS mostly focus on single mechanisms and lack a systematic understanding of central–peripheral immune interactions (Yu et al., 2022). This has limited the translational application of novel diagnostic biomarkers and therapeutic strategies.

Lipocalin-2 (LCN2) is a typical inflammatory response factor, which has been found to be closely associated with several neurodegenerative diseases in recent years. It has been shown that LCN2 is upregulated in Alzheimer’s disease (AD), Parkinson’s disease (PD), and multiple sclerosis (MS), and is involved in pathological processes through mechanisms such as neuroinflammation, cell death signaling pathways, and iron metabolism disorders (Kang et al., 2021; Fan et al., 2024; Berard et al., 2012). Considering that ALS is also characterized by significant inflammation and metabolic imbalance (Feldman et al., 2022). We hypothesize that LCN2 may serve as a key molecular link connecting neuroinflammation, iron homeostasis imbalance, and immune dysregulation. By influencing both central and peripheral immune microenvironments, it may heighten the vulnerability of the nervous system to harmful stimuli. LCN2 may play a significant role in the onset and progression of ALS.

In this review, we first introduce the structural characteristics and physiological functions of LCN2, followed by a summary of its expression alterations and upregulation mechanisms in ALS. Subsequently, we focus on elucidating the role of LCN2 in pathological processes such as neuroinflammation, iron dysregulation, cell death, and peripheral immune immunity. We propose a central-peripheral linkage hypothesis mediated by LCN2 to explain its potential role in the pathogenesis of ALS. Finally, we explore the clinical application prospects of LCN2, including its potential as a biomarker and its value and challenges as a therapeutic target. Through systematic integration and critical evaluation of existing research, this review aims to reveal the key role of LCN2 in ALS and propose its potential applications in advancing precision diagnosis and targeted therapy.

## 2 Structure and physiological roles of LCN2

LCN2, also known as neutrophil gelatinase-associated lipocalin, was originally identified in the neutrophil gelatinase subcellular compartment at sites of infection and inflammation (Flower et al., 2000; Xiao et al., 2017). The *LCN2* gene is located on chromosome 9 at the 9q34.11 locus (Jaberi et al., 2021). It is a secreted protein containing 198 amino acids with a molecular weight of approximately 25 kDa. LCN2 is a member of the lipocalin superfamily, which includes over 20 small secreted proteins. These proteins are involved in transporting and regulating various hydrophobic small molecules, such as vitamins, steroids, lipids, and iron carriers (Dekens et al., 2021). Structurally, lipocalins share a common feature: a barrel-like fold formed by eight antiparallel *β*-strands (Flower, 1994). This forms a hydrophobic cavity that binds ligands. The specificity of ligand binding varies among family members due to differences in the amino acid residues lining this cavity (Flower et al., 2000; Flower, 1996; Flower, 2000). Compared to other lipocalins, LCN2 has a larger and more polar cavity than most other members. This enables it to bind larger and less hydrophobic ligands, including lipopolysaccharides and mammalian proteins. This allows LCN2 to form macromolecular receptor complexes, contributing to its diverse roles in immunomodulation and cellular regulation (Bao et al., 2015b; Goetz et al., 2000; Goetz et al., 2002). Moreover, LCN2 functions as an iron-binding protein that sequesters bacterial siderophores, thereby limiting microbial access to iron—a key mechanism in host defense (Bachman et al., 2012; Guo et al., 2020). It also plays an essential role in maintaining iron homeostasis in mammals (Bao et al., 2015a,b; Bao et al., 2010; Bao et al., 2013).

LCN2 enters cells by binding to specific receptors. The two main receptors are 24p3R and megalin (Devireddy et al., 2005; Hvidberg et al., 2005). The 24p3R receptor can also bind albumin, metallothionein, plant chelators, and possibly transferrin (Dizin et al., 2013; Langelueddecke et al., 2012; Langelueddecke et al., 2014). Megalin binds several ligands, including albumin, insulin, insulin-like growth factor 1, matrix metalloproteinase 9 (MMP9), and hemoglobin (Marzolo and Farfán, 2011; Van den Steen et al., 2006). Multiple splice variants of 24p3R exist. All can act as functional receptors for LCN2, though with different binding strengths (Devireddy et al., 2005; Fang et al., 2007). Recent studies show that LCN2 can also bind to melanocortin 4 receptor (Mosialou et al., 2017). It may also activate melanocortin 1 receptor and melanocortin 3 receptor (Mosialou et al., 2017). These findings suggest that LCN2 is a potential ligand in the melanocortin signaling pathway. Other ligands in this system include *α*-melanocyte-stimulating hormone, *β*-melanocyte-stimulating hormone, adrenocorticotropic hormone, and its antagonist agouti-related protein (Tao, 2010). Although the exact binding sites remain unclear, computational studies have predicted possible interaction regions. LCN2 participates in many physiological processes. These include defense against bacterial infection, regulation of iron metabolism, control of cell death and survival signals, and modulation of inflammation. LCN2 also promotes chemotaxis, cell migration, and differentiation. It helps regulate energy metabolism (Flo et al., 2004; Jha et al., 2015; Chakraborty et al., 2012; Cakal et al., 2011; Noçon et al., 2014). Disruptions in these functions may result from, or lead to, altered LCN2 expression or receptor signaling. Therefore, LCN2 plays an important role in maintaining homeostasis.

In the periphery, LCN2 is expressed at low levels and shows strong cell specificity in healthy adults. It is present in neutrophils, bone marrow, osteoblasts, fat tissue, the heart, blood vessels, uterus, prostate, and salivary glands. It is also found in epithelial cells of the respiratory, urinary, and digestive systems (Hvidberg et al., 2005; Mosialou et al., 2017; Borregaard and Cowland, 2006; Cowland and Borregaard, 1997; Devarajan, 2007; Wang et al., 2007; Yndestad et al., 2009; Zhang et al., 2012). Neutrophils store LCN2 for rapid release, and epithelial cells maintain sustained low expression. These features help mount a quick immune response. In muscle and fat tissue, LCN2 also regulates glucose and insulin balance, appetite, and iron metabolism (Mosialou et al., 2017; Abella et al., 2015; Nairz et al., 2009). The receptors for LCN2, including megalin and 24p3R, are widely distributed in the body (Devireddy et al., 2005; Hvidberg et al., 2005; Jha et al., 2015; Chia et al., 2015). This indicates that LCN2 signaling has systemic effects. In contrast, MC4R is mainly expressed in the CNS (Tao, 2010).

In the CNS, LCN2 is expressed at low levels under normal conditions (Chia et al., 2011; Dekens et al., 2018; Hovens et al., 2016). Its expression increases significantly in neurological diseases such as stroke, AD, and PD. In these conditions, LCN2 is strongly associated with inflammation, iron imbalance, and neuron injury. For example, blood and cerebrospinal fluid levels of LCN2 increase with stroke severity (Zhao et al., 2023). In PD, plasma LCN2 levels correlate with non-motor symptoms and iron accumulation in the substantia nigra (Kang et al., 2021). In AD, LCN2 is upregulated in the frontal cortex and may contribute to cognitive decline through its effects on inflammation and iron homeostasis (Fan et al., 2024; Eidson et al., 2017; Xiong et al., 2022). These findings support a conserved and broad role for LCN2 in neurodegeneration. LCN2 may serve as both a biomarker and a contributor to disease progression.

Although research on LCN2 in ALS is still limited, early findings suggest it plays a role in disease pathogenesis. LCN2 has emerged as a potential diagnostic marker for ALS, based on the observation of elevated LCN2 in the plasma of patients with ALS. One clinical study involving 68 ALS patients and 34 healthy controls showed significantly elevated plasma LCN2 levels in the ALS group (Ngo et al., 2015). Similar results were seen in another independent study (Petrozziello et al., 2020). However, no significant relationship was found between LCN2 levels and ALS functional rating scale revised scores or disease duration in these patients. This may be attributable to the lack of stratification for factors such as site of onset, disease progression rate, and pathological subtypes, which could obscure potential associations with functional scores or disease course. In addition, LCN2 levels may be significantly elevated only at specific time points during disease progression, and single-time-point sampling in these studies may have missed the optimal window to detect such associations. Despite these limitations, these studies provide the most direct clinical evidence to date, although sample sizes remain modest. Based on these findings, future studies may require more precise patient selection and longitudinal monitoring of LCN2 levels. Therefore, clinicians should exercise caution when considering LCN2 levels as a clinical biomarker for ALS. Tissue studies support these findings. Brain and spinal cord samples from ALS patients showed increased LCN2 expression in the motor cortex and spinal cord. However, 24p3R levels did not differ significantly between patients and controls (Petrozziello et al., 2020). Taken together, these results indicate elevated LCN2 expression in ALS, but its precise spatial and cellular localization will likely require advanced technologies such as spatial transcriptomics for clarification in future studies. Genetic analyses using the ALS Knowledge Portal and Project MinE databases identified several variants in the LCN2 gene that may be related to ALS risk (Petrozziello et al., 2020). These are database-based predictions, lacking functional experimental validation. Animal studies provide further support. In ALS rat models carrying TAR DNA-binding protein 43 (TDP-43) or fused in sarcoma (FUS) mutations, activated astrocytes released LCN2. This led to selective toxicity in motor neurons (Tong et al., 2013; Bi et al., 2013; Huang et al., 2014). Based on animal models, these findings suggest a potential mechanism, but whether this applies to human patients remains unclear. These results suggest that LCN2 plays a role in the astrocyte–motor neuron interaction. In summary, although LCN2 research in ALS is still at an early stage, clinical samples, tissue analyses, genetic data, and animal models all support its involvement in disease development. This review aims to summarize current findings, explore the mechanisms behind LCN2 upregulation in ALS, and provide a basis for future therapeutic strategies.

## 3 Mechanisms of LCN2 upregulation

Previous studies have shown that LCN2 is significantly upregulated in both the CNS and peripheral blood of patients with ALS compared with healthy controls. This suggests that LCN2 may play an important role in disease development. However, the mechanisms driving its upregulation differ between central and peripheral tissues. In the CNS, LCN2 is mainly produced by astrocytes and regulated by various inflammation-related signaling pathways (Tong et al., 2013; Bi et al., 2013; Huang et al., 2014). In the periphery, neutrophils are the primary source, and LCN2 secretion is influenced by inflammatory stimuli and degranulation processes (Kjeldsen et al., 1994). The following sections summarize the cellular sources and molecular mechanisms responsible for LCN2 upregulation in both compartments.

### 3.1 LCN2 upregulation mechanisms in the CNS

**Figure 1:**
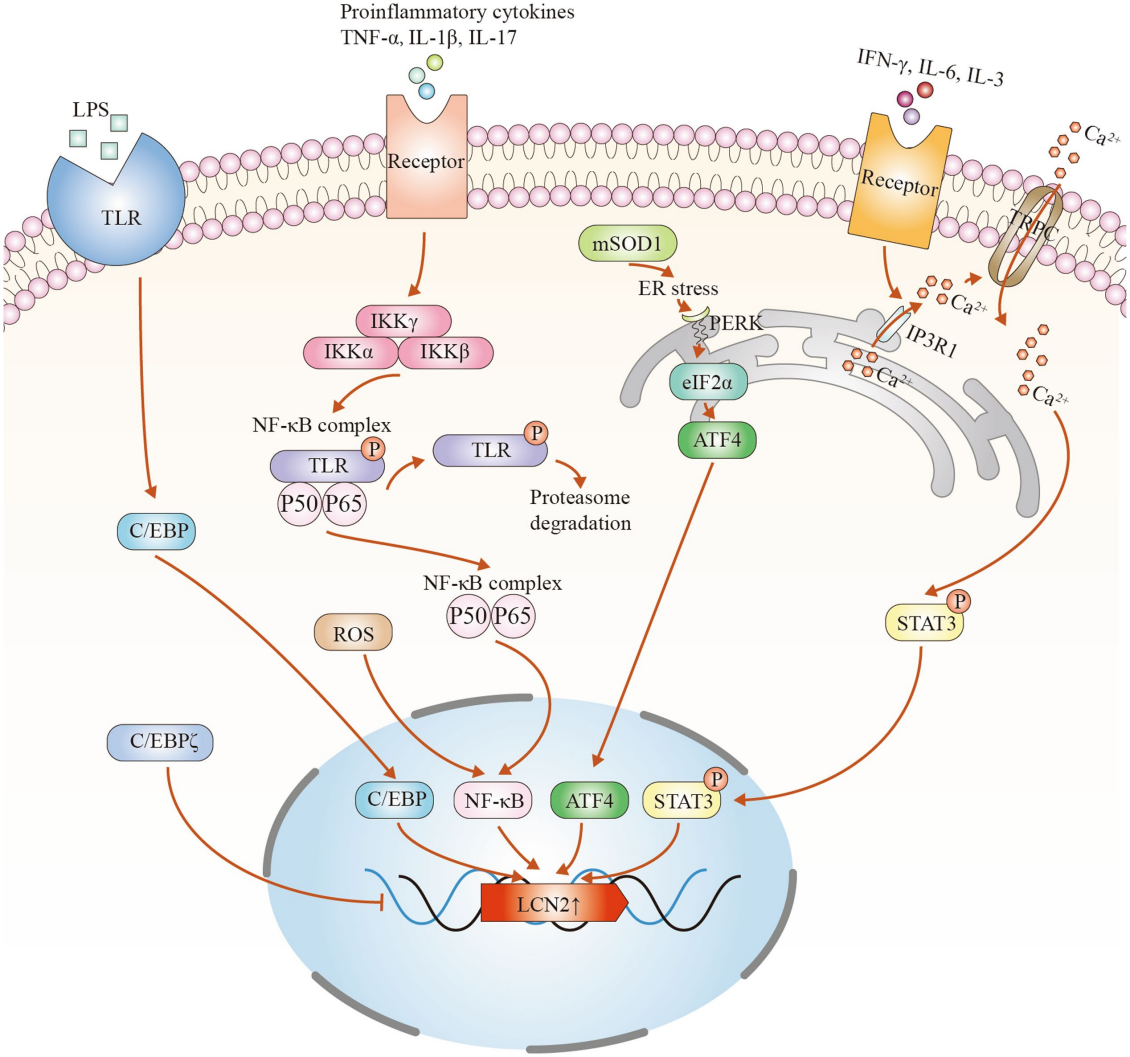
Potential mechanisms of LCN2 upregulation in amyotrophic lateral sclerosis (ALS). (1) Inflammatory mediators such as lipopolysaccharide (LPS) bind to cell surface receptors like Toll-like receptors (TLRs), activating the CCAAT/enhancer-binding protein (C/EBP) signaling pathway and inducing LCN2 transcription. C/EBPζ negatively regulates lipocalin-2 (LCN2) expression. (2) Pro-inflammatory cytokines activate signaling cascades that lead to ubiquitin–proteasome-mediated degradation of nuclear factor-κB inhibitor *α* (NFKBIA/IκBα), allowing the nuclear factor κB (NF-κB) complex to translocate into the nucleus and promote LCN2 transcription. (3) Aggregation of mutant superoxide dismutase 1 (SOD1) induces endoplasmic reticulum (ER) stress and activates the protein kinase R-like endoplasmic reticulum kinase–eukaryotic initiation factor 2 alpha–activating transcription factor 4 (PERK–eIF2α–ATF4) pathway, increasing LCN2 expression. (4) Pro-inflammatory factors stimulate inositol 1,4,5-trisphosphate receptor type 1 (IP3R1) on the cell membrane, triggering ER Ca^2+^ release. Subsequent activation of transient receptor potential canonical (TRPC) channels sustains intracellular Ca^2+^ influx, maintaining signal transducer and activator of transcription 3 (STAT3) phosphorylation and promoting LCN2 transcription.

In the CNS, LCN2 can be expressed by a variety of cell types, including neurons, microglia, astrocytes and endothelial cells. However, in ALS, astrocytes are thought to be the main source of LCN2 expression (Tong et al., 2013; Bi et al., 2013; Huang et al., 2014). Several signaling pathways contribute to its upregulation. Key transcription factors include nuclear factor-κB (NF-κB), CCAAT/enhancer-binding protein (C/EBP), signal transducer and activator of transcription 3 (STAT3), and activating transcription factor 4 (ATF4) (Figure 1). Among these, the NF-κB pathway plays a central role. Inflammatory stress, such as lipopolysaccharide stimulation, activates NF-κB in a manner that mimics glial activation in neurodegenerative diseases. Damaged neurons or inflammatory cues first activate microglia, which release pro-inflammatory factors. These then trigger NF-κB signaling in astrocytes. Specifically, degradation of IκBα allows the p50/p65 complex to translocate to the nucleus and initiate LCN2 transcription (Jaberi et al., 2021; Jung et al., 2023). Oxidative stress can also enhance this pathway (Liu et al., 2022). Second, the C/EBP family is another important regulator. Inflammatory stimuli activate this pathway to increase LCN2 expression, whereas deficiency of C/EBP significantly reduces LCN2 levels (Jha et al., 2015). Interestingly, C/EBPζ acts as a negative regulator and may inhibit LCN2 expression by interfering with NF-κB binding at promoter sites (Wang L. et al., 2011). Third, the STAT3 pathway is also involved. Cytokines such as interleukin-3, interleukin-6, and interferon-*γ* activate inositol trisphosphate receptor type 1 in the astrocytic endoplasmic reticulum. This promotes calcium release and activates the cation channel transient receptor potential canonical (TRPC), allowing continuous calcium influx. Sustained calcium signaling maintains STAT3 phosphorylation, which initiates LCN2 transcription (Shiratori-Hayashi et al., 2021). Notably, this pathway has been found to be activated in the spinal cord of patients with sporadic ALS (Shibata et al., 2010). Finally, the Protein Kinase RNA-like Endoplasmic Reticulum Kinase (PERK)-eukaryotic Initiation Factor 2α (eIF2α)-ATF4 signaling pathway, which is associated with endoplasmic reticulum stress, has been shown to induce LCN2 expression in astrocytes (Wang et al., 2024). In ALS models, misfolded superoxide dismutase 1 (SOD1) aggregates trigger the unfolded protein response, thereby activating this pathway (Dzhashiashvili et al., 2019). However, whether this directly promotes LCN2 expression in human ALS patients requires further investigation. In summary, LCN2 upregulation in the CNS of ALS involves multiple signaling pathways and transcription factors. Astrocytes are the key cellular responders. Understanding these molecular mechanisms will help clarify the role of LCN2 in ALS pathogenesis and may guide future therapeutic approaches.

### 3.2 LCN2 upregulation mechanisms in peripheral tissues

In addition to the CNS, LCN2 levels are also significantly elevated in the peripheral blood of ALS patients. Peripheral LCN2 is secreted by several immune and non-immune cell types, including neutrophils, adipocytes, and hepatocytes. Among these, neutrophils are considered the main source (Kjeldsen et al., 1994). Neutrophil counts are often increased in ALS patients and show a positive correlation with disease progression (Murdock et al., 2017; Wang et al., 2025; Murdock et al., 2021; Jiang et al., 2024). LCN2 was first discovered in neutrophils, where it is synthesized during early maturation and stored in cytoplasmic granules (Ferreira et al., 2015). Neutrophils contain three types of granules: primary (azurophilic), secondary (specific), and tertiary (gelatinase) granules. LCN2 is mainly stored in secondary granules, which also participate in extracellular matrix degradation and immune regulation (Zhang et al., 2022). During tissue injury, infection, or inflammation, these granules release their contents either to the cell surface or into the extracellular space through a tightly regulated exocytosis process. Although the exact mechanism of neutrophil degranulation remains unclear, SNARE proteins and Rab GTPases are thought to play essential roles (Mollinedo, 2019). LCN2 also forms a complex with MMP9, which stabilizes MMP9 and prevents its self-degradation (Zhao et al., 2023; Kubben et al., 2007; Tschesche et al., 2001). However, when LCN2 undergoes polyamidation or deamidation, its ability to form this complex decreases. As a result, MMP9 is degraded more rapidly. While this mechanism sheds light on the potential role of LCN2 in inflammation and matrix remodeling, its impact on LCN2 stability and biological activity is still unclear (Jung and Ryu, 2023). Taken together, current studies have identified neutrophils as the primary source of peripheral LCN2 in ALS and have begun to reveal the mechanisms regulating its synthesis and release. Future research should aim to clarify how LCN2 production and function are regulated in neutrophils and how these processes contribute to disease progression. A deeper understanding may provide new targets for therapeutic intervention in ALS.

## 4 Role of LCN2 in ALS

**Figure 2:**
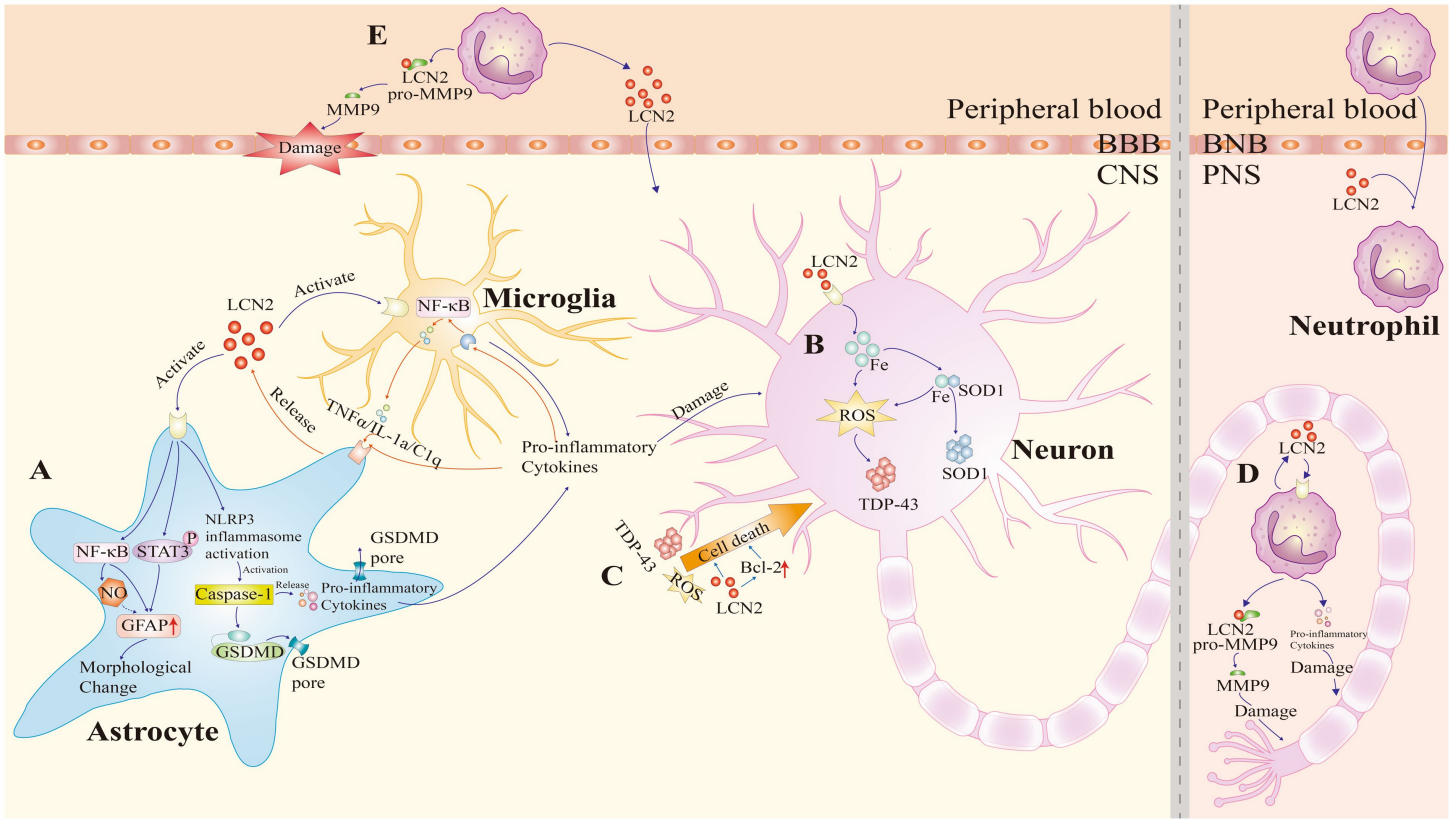
Pathogenic roles of lipocalin-2 (LCN2) in amyotrophic lateral sclerosis (ALS). LCN2 contributes to ALS pathogenesis through four major mechanisms: neuroinflammation, iron dysregulation, cell death, and peripheral immunity. **(A)** Neuroinflammation: LCN2 binds to cell surface receptors on astrocytes, activating the nuclear factor-κB (NF-κB) and Janus kinase/signal transducer and activator of transcription 3 (JAK/STAT3) signaling pathways, and promoting nitric oxide (NO) production, which upregulates glial fibrillary acidic protein (GFAP) expression. This induces morphological changes in astrocytes. LCN2 also activates the NOD-like receptor family pyrin domain containing 3 (NLRP3) inflammasome, leading to GSDMD-mediated membrane pore formation and pro-inflammatory cytokine release. In microglia, LCN2 enhances the release of inflammatory mediators. Additionally, pro-inflammatory factors activate the NF-κB signaling pathway, exacerbating neuroinflammation. This forms a positive feedback loop that further promotes astrocytic LCN2 expression. **(B)** Iron dysregulation: LCN2 promotes intracellular iron accumulation, which facilitates the aggregation of superoxide dismutase 1 (SOD1). Additionally, iron accumulation generates reactive oxygen species (ROS), which in turn promotes the aggregation of TAR DNA-binding protein 43 (TDP-43). **(C)** Cell death: LCN2 directly upregulates Bcl-2 family proteins to promote apoptosis. Additionally, inflammatory damage, ROS generation, and mutant TDP-43 increase motor neuron vulnerability to LCN2-induced toxicity, ultimately leading to neuronal death. **(D,E)** Peripheral immunity: upon stimulation, neutrophils release LCN2 and pro-inflammatory cytokines, causing peripheral nerve injury. LCN2 also activates matrix metalloproteinase-9 (MMP9), resulting in axonal damage, disruption of the blood–brain barrier (BBB), and amplification of local inflammatory responses. BNB, blood-nerve barrier; CNS, central nervous system; PNS, peripheral nervous system.

LCN2 is upregulated in both the CNS and peripheral blood of patients with ALS, suggesting its potential role in both compartments. This section explores how LCN2 contributes to ALS pathogenesis through four key mechanisms: neuroinflammation, iron metabolism, cell death, and peripheral immunity (Figure 2).

### 4.1 Neuroinflammation

Neuroinflammation helps clear pathogens, promote tissue repair, and remove cellular debris. However, when persistent, it can suppress tissue regeneration and drive disease progression (Gaudet and Fonken, 2018; Wyss-Coray and Mucke, 2002). In ALS, neuroinflammation is a common pathological feature. It typically presents as the activation and infiltration of glial cells (Beers and Appel, 2019). Recent single-cell studies further reveal that ALS can be classified into oxidative stress-driven, glial activation-driven, and TDP-43 activation-driven subtypes. These molecular subtypes manifest in both neurons and glial cells and are closely associated with disease progression (O'Neill et al., 2025). Concurrently, single-cell transcriptomic data from ALS patients’ brains and spinal cords indicate that microglia and astrocytes exhibit widespread pro-inflammatory and activated states (Zelic et al., 2025). Specifically, interferon-responsive microglia are enriched in patients with accelerated disease progression, while protective microglial subpopulations are significantly depleted (Tuddenham et al., 2025). This further underscores the central role of glial dysregulation in ALS neuroinflammation and disease progression. Glial cells—such as microglia, astrocytes, and oligodendrocytes—were once seen as structural supporters of neurons. Recent studies reveal that they also have immune-modulatory functions (Tan et al., 2024; Somjen, 1988; Greenhalgh et al., 2020; Allen and Lyons, 2018). Among them, microglia and astrocytes are the main drivers of neuroinflammation in ALS. Microglia are resident macrophages of the CNS and account for 5–15% of brain cells. They are responsible for immune surveillance and inflammatory responses. Based on their activation state, they are categorized into pro-inflammatory M1 and anti-inflammatory M2 types (Allen and Lyons, 2018). Astrocytes, the most abundant glial cells in the brain, also show functional diversity. Type A1 astrocytes are pro-inflammatory and are the primary source of LCN2 (Suk, 2016). In contrast, type A2 astrocytes secrete anti-inflammatory factors that support repair (Liddelow and Barres, 2017; Liddelow et al., 2017). Microglia and astrocytes interact closely. During inflammation, they amplify each other’s activation via positive feedback loops (Tan et al., 2024). This functional imbalance promotes disease progression. Thus, dysfunctional glial responses are a core driver of neuroinflammation in ALS.

LCN2 is a typical inflammation-associated protein that regulates the level of inflammation through multiple mechanisms. In the ALS rat model, it was found that LCN2 is mainly produced by reactive astrocytes. And it can bind to megalin and 24p3R receptors on the surface of astrocytes and microglia, inducing the release of pro-inflammatory factors, thus driving the development and maintenance of neuroinflammation (Tong et al., 2013; Bi et al., 2013; Huang et al., 2014). Therefore, LCN2 is recognized as a key mediator in neuroinflammatory responses. Although most studies suggest that LCN2 promotes the pro-inflammatory activation of glial cells (Dekens et al., 2021), contradictory results have also been reported. For instance, in J20 transgenic mice, knockout of LCN2 did not alter glial cell activation (Dekens et al., 2018). In another study, LCN2-deficient mice challenged with lipopolysaccharide exhibited strong neuroprotective effects, indicating a potential anti-inflammatory role of LCN2 (Kang et al., 2018). The reasons for these contradictory findings remain unclear. They may be attributed to differences in the specific CNS disease models employed, the experimental protocols used, the disease stages investigated, or the cell types involved. Future studies will be needed to clarify the precise role of LCN2 under these varying conditions. Moreover, the exact mechanisms underlying its potential immunomodulatory functions have yet to be fully elucidated.

As the main source of LCN2, astrocytes not only regulate its secretion, but also further activate themselves through the autocrine effect of LCN2. The specific mechanisms are as follows. First, LCN2 is secreted and then binds to the receptor via the auto−/paracrine pathway, activating the NF-κB signaling pathway and the Janus kinase (JAK)/STAT3 pathway and nitric oxide (NO) production. It can upregulate glial fibrillary acidic protein (GFAP) expression and induce astrocyte polarization, morphological changes, and glial scar formation (Jung and Ryu, 2023; Lee et al., 2009; Wang et al., 2021; Zhao et al., 2019). Second, LCN2 upregulation activates NLRP3 inflammatory vesicles, which promote GSDMD-mediated membrane pore formation and release pro-inflammatory factors. This further exacerbates neuroinflammation (Li J. et al., 2023). It was also found that LCN2 gene silencing by adeno-associated virus significantly reduced A1 astrocytes and increased A2 cells, thereby alleviating neuroinflammation and promoting blood–brain barrier (BBB) recovery (Xiao et al., 2025). These findings suggest that LCN2 is both a product and driver of astrocyte activation. In addition to astrocytes, LCN2 can significantly affect microglia function. In the ALS model, the high expression of LCN2 receptors on microglia suggests their high sensitivity to LCN2 (Bi et al., 2013). LCN2 promotes microglial polarization toward the M1 phenotype (Xiang et al., 2022). In turn, these M1 microglia release pro-inflammatory cytokines such as tumor necrosis factor-*α* (TNF-α) and interleukin-1α (IL-1α), which induce the conversion of astrocytes into the A1 phenotype (Jung and Ryu, 2023). This feedback loop intensifies neuroinflammation. In other neurological disease models, such as surgery-induced neuroinflammation or post-stroke depression models (Xiang et al., 2022; Wei et al., 2021), it has also been observed that LCN2 can modulate the inflammatory transformation of microglia and thus influence the degree of inflammation. Ultimately, LCN2-activated astrocytes and microglia together release a large number of proinflammatory factors and chemokines, creating an amplified effect of local inflammation (Schröder et al., 2023; Loane and Byrnes, 2010; Nakagawa and Chiba, 2015). These factors damage the BBB and attract peripheral immune cells into the brain, leading to neuronal injury (Liddelow et al., 2017). Together, they form a feedback loop of inflammation, activation, and damage centered on glial cells. This loop likely plays a key role in driving ALS progression.

In summary, neuroinflammation is central to the pathogenesis of ALS. LCN2 acts as a key molecule linking astrocytes and microglia. Targeting LCN2 and its signaling pathways may offer new strategies for treating ALS and other neurodegenerative diseases.

### 4.2 Iron dysregulation

Iron has an irreplaceable role in numerous biological processes, including cell division, oxygen transport, maintenance of mitochondrial function, synaptic development, myelin formation, and neurotransmitter metabolism (Beard and Connor, 2003). In living organisms, iron mainly functions through redox cycling between Fe^2+^ and Fe^3+^, supporting electron transfer reactions. However, in pathological conditions, iron imbalance can lead to excessive production of reactive oxygen species (ROS), resulting in oxidative stress. This process has been recognized as a key pathophysiological mechanism in neurodegenerative diseases (Hare et al., 2013).

Numerous clinical and experimental studies have confirmed abnormal iron metabolism in ALS. Neuroimaging using 3 T and 7 T magnetic resonance imaging has shown iron deposition in cortical regions associated with symptom onset (Zejlon et al., 2024). Susceptibility-weighted imaging often reveals hypointense signals in the motor cortex, indicating iron accumulation (Zejlon et al., 2024). This imaging feature is now considered a diagnostic marker in ALS and is frequently used in clinical trials. Autopsy studies have also confirmed significant deposits of trivalent iron in the anterior central gyrus of ALS patients (Petillon et al., 2018). Furthermore, a meta-analysis showed that serum ferritin levels are significantly higher in ALS patients compared to healthy controls (Petillon et al., 2018). Similar findings have been reported in transgenic SOD1 mutant mice (e.g., G91A, G37R), in which elevated spinal cord iron levels were associated with altered expression of iron-related proteins (Moreau et al., 2018). Iron chelation therapy has shown potential benefits. In SOD1 mutant mice, treatment with the iron chelator deferiprone prolonged survival. In a clinical study involving 23 ALS patients, deferiprone reduced iron accumulation in the cervical cord, medulla oblongata, and motor cortex. It also lowered oxidative stress markers and neurofilament light chain levels in cerebrospinal fluid (Moreau et al., 2018). These findings suggest that iron overload contributes to ALS pathogenesis and progression. Mechanistically, iron accumulation promotes the misfolding and aggregation of disease-related proteins, such as mutant SOD1 and TDP-43, through oxidative stress (Joppe et al., 2019). Iron can bind directly to SOD1 and enhance its aggregation (Lim and Song, 2015). In SOD1 mutant models, iron-induced ROS production leads to TDP-43 aggregation (Cohen et al., 2012), which can be reduced by iron chelators (Wang Q. et al., 2011). Therefore, iron catalyzes the Fenton reaction, generating ROS that cause oxidative modifications to proteins (e.g., carbonylation, aberrant disulfide bond formation), which further disrupt protein structure and result in toxic aggregates that damage motor neurons.

LCN2 is a critical regulator of iron homeostasis and may serve as a link between iron dysregulation and neuronal injury. It binds to iron-loaded or iron-free siderophores and mediates iron transport across cell membranes (Xiao et al., 2017). When bound to iron, LCN2 enters the cytosol and increases intracellular iron content. When iron-free, it removes excess iron from the cell, reducing intracellular iron levels (Richardson, 2005). In LCN2-deficient mice, increased brain iron and oxidative stress further highlight its role in maintaining iron balance (Nairz et al., 2009; Dekens et al., 2018; Ferreira et al., 2018; Nairz et al., 2015).

LCN2 is also involved in iron metabolism in other neurodegenerative diseases. In AD models, inhibition of LCN2 reduced hippocampal iron accumulation (especially in plaques and hippocampal pyramidal and granular neurons) (Dekens et al., 2018). However, another study reported that in AD, LCN2 failed to regulate iron accumulation in astrocytes (Dekens et al., 2020). In PD models, LCN2 mediated iron-induced dopaminergic neuron damage, which was mitigated by iron chelation (Kim et al., 2016). In MS, cerebrospinal fluid LCN2 levels correlated with iron accumulation in the basal ganglia (Khalil et al., 2016). LCN2 upregulation has also been observed alongside iron accumulation in models of intracerebral hemorrhage and ischemic stroke (Hochmeister et al., 2016). regulates brain iron export by influencing hepcidin and ferroportin expression. As a transporter, it can also directly mediate cell-to-cell iron exchange. LCN2 deficiency impairs cellular iron export, reinforcing its essential role in iron metabolism (Lim et al., 2021). It should be noted that the above studies suggest that iron dysregulation by LCN2 may exhibit cell type and disease specificity. Although direct evidence for LCN2’s role in ALS iron regulation is lacking, findings from AD and PD models offer important clues. In AD, LCN2 knockdown corrected iron-related gene expression abnormalities and reduced *β*-amyloid-induced iron imbalance (Dekens et al., 2018). In PD, LCN2 accumulation is considered part of disease progression (Kim et al., 2016; Lee et al., 2012; Ward et al., 2014). Given that protein aggregation is a shared feature of neurodegenerative diseases, we hypothesize that LCN2 upregulation in ALS may worsen protein aggregation by disrupting iron homeostasis, thereby accelerating motor neuron degeneration.

In conclusion, iron dysregulation plays a key role in ALS pathogenesis. LCN2 may contribute to neuronal injury by disrupting iron metabolism, enhancing oxidative stress, and promoting protein aggregation. Further studies are needed to verify this hypothesis and explore LCN2 as a potential therapeutic target in ALS.

### 4.3 Cell death

In ALS, cell death is the core pathological endpoint that determines the rate of motor neuron loss and disease progression (Thal et al., 2024). In various neurodegenerative diseases, LCN2 is widely recognized as a key mediator of cell death (Dekens et al., 2021). It can influence the susceptibility of CNS cells—such as neurons, astrocytes, and microglia—to harmful stimuli. LCN2 promotes apoptosis not only through direct mechanisms but also by modifying the extracellular environment, inducing inflammation, or disrupting metal ion homeostasis (Dekens et al., 2021). In ALS, increased LCN2 expression is closely linked to disease progression, suggesting its involvement in multiple aspects of pathogenesis (Petrozziello et al., 2020). The following section reviews current evidence on LCN2-mediated cytotoxicity in ALS.

In ALS models, LCN2 shows selective toxicity to neurons expressing ALS-associated genes such as mutant FUS or TDP-43 (Tong et al., 2013; Bi et al., 2013). *In vitro* studies have confirmed that LCN2-containing conditioned medium induces neuronal death in primary cultures and brain slices. Removal of LCN2 from the medium significantly reduces this toxicity, indicating its role as a key effector (Petrozziello et al., 2020; Bi et al., 2013). One known mechanism involves LCN2-mediated upregulation of pro-apoptotic Bcl-2 family proteins, such as Bim (Devireddy et al., 2005; Lee et al., 2009). Silencing Bim partially reverses the pro-apoptotic effect, although other pathways may also be involved (Lee et al., 2007; Naudé et al., 2012). LCN2 not only acts directly on neurons but also contributes to indirect toxicity by modulating glial cells. It promotes astrocyte and microglial activation and stimulates the release of pro-inflammatory cytokines such as IL-1β and TNF-*α*, leading to enhanced local inflammation. This, in turn, increases neuronal vulnerability to death signals (Li J. et al., 2023; Mesquita et al., 2014). In the context of ALS, mutant TDP-43 has been shown to induce astrocytic LCN2 release, which further sensitizes motor neurons to toxic stimuli, contributing to non-cell-autonomous mechanisms of neuronal death (Jha et al., 2015; Tong et al., 2013; Bi et al., 2013; Huang et al., 2014). Interestingly, LCN2 may also mediate neuronal apoptosis through iron-related mechanisms in certain neurological diseases (Dekens et al., 2021), direct validation in ALS remains lacking. However, in ALS-specific studies, iron and transferrin do not appear to significantly influence LCN2-induced neuronal toxicity (Bi et al., 2013). This discrepancy suggests that LCN2 may operate through disease-specific mechanisms. This suggests that LCN2-mediated neuronal death may be disease-specific and could also be associated with differences in experimental methods or detection indicators.

Although LCN2 shows significant toxicity to neurons in ALS, its direct effect on glial cell survival appears limited. LCN2 does not typically induce astrocyte or microglial death but can cause morphological and functional changes that increase their sensitivity to external stressors such as NO (Bi et al., 2013; Lee et al., 2009; Lee et al., 2007). In microglia, LCN2 increases apoptotic susceptibility. This enhanced vulnerability may relate to disruptions in iron metabolism, although it does not seem to involve changes in Bcl-2 family protein expression (Lee et al., 2007). Therefore, the cytotoxicity of LCN2 in ALS is likely to be cell type–specific.

In summary, LCN2 contributes to cell death in ALS through both direct and indirect mechanisms. It can directly trigger neuronal apoptosis by upregulating pro-apoptotic genes and indirectly exacerbate neurodegeneration by activating glial cells and promoting inflammation. These effects may vary by cell type, indicating that LCN2 could influence ALS progression through cell-specific pathways. Although several studies highlight the importance of LCN2 in ALS-related cytotoxicity, the full scope of its molecular mechanisms remains unclear. Further research is needed to define its precise role and to evaluate its potential as a therapeutic target in ALS. However, most existing evidence remains at the stage of indirect inference, lacking direct validation in the neural tissue of ALS patients. Therefore, further confirmation of its molecular mechanisms requires integrating human multi-omics and single-cell level studies.

### 4.4 Peripheral immunity

Studies have shown that LCN2 also plays a key role in the peripheral immune system, being particularly crucial in regulating neutrophil activity and inflammatory responses (Kjeldsen et al., 1994). It is particularly important in regulating neutrophil activity and inflammatory responses. Compared with classic neurodegenerative diseases such as AD and PD, the pathogenesis of ALS remains less clear. One unresolved question is the exact site of disease initiation (Kiernan et al., 2011). Traditionally, ALS has been considered a centrally driven disease, beginning in the CNS and spreading to peripheral nerves and neuromuscular junctions (NMJ) in a paracrine manner (Menon et al., 2015). However, growing evidence suggests an alternative possibility: motor neuron degeneration may begin at NMJ and progress retrogradely to the cell bodies (Verma et al., 2022; Dadon-Nachum et al., 2011). Our group studied 112 ALS patients within 1 year of disease onset. We found that peripheral axonal damage was closely associated with a faster rate of disease progression, indicating that pathological changes in the peripheral nervous system may emerge early in the disease course (Yu et al., 2021). Further research has shown that neutrophils may contribute to ALS progression by promoting peripheral nerve injury (Wang et al., 2025). These findings highlight the potential importance of the peripheral immune system in ALS and suggest that the “peripheral pathology” of ALS is distinct from other neurodegenerative diseases. This offers a new entry point for exploring ALS pathogenesis.

LCN2 is a major secretory protein of neutrophils and plays a crucial role in peripheral immune regulation. In human neutrophils, LCN2 is stored in secondary granules and can be rapidly released upon exposure to inflammatory stimuli (Kjeldsen et al., 1994). Once secreted, LCN2 binds to the receptor on immune cells, promoting the production of pro-inflammatory cytokines such as TNF-*α*, IL-1β, and IL-8. Through autocrine and paracrine signaling, it activates neutrophils and other inflammatory cells, thereby sustaining local inflammation (Jha et al., 2015). However, in ALS, the secretion characteristics of LCN2 in peripheral neutrophils remain poorly understood, and future research should employ technologies such as single-cell sequencing for in-depth analysis. In addition, LCN2 can form a complex with MMP9, a zinc-dependent protease that degrades extracellular matrix components. This complex inhibits MMP9 self-degradation and enhances its enzymatic activity (Amersfoort et al., 2018). Multiple studies have reported elevated serum MMP9 levels in ALS patients, which are associated with faster disease progression (Amersfoort et al., 2018). Moreover, certain MMP9 gene polymorphisms are linked to increased ALS risk (He et al., 2013). LCN2 may contribute to peripheral pathology in ALS through the following MMP9-dependent mechanisms: (1) Axonal injury: MMP9 disrupts NMJ architecture, leading to preferential degeneration of fast motor neurons (Spiller et al., 2019). (2) BBB disruption: Neutrophil-derived MMP9 increases BBB permeability, facilitating peripheral immune cell infiltration into the CNS (Gidday et al., 2005). (3) Inflammatory amplification: MMP9 promotes the proteolytic activation of inflammatory cytokines like TNF-α, further aggravating neuroinflammation and neuronal apoptosis (Kiaei et al., 2007).

In summary, LCN2 is not only a key mediator of central neuronal injury in ALS but also contributes significantly to peripheral nerve damage. It exerts these effects by regulating neutrophil function, enhancing MMP9 activity, and promoting the breakdown of NMJs and the BBB.

### 4.5 Mechanistic circuit model: central-peripheral linkage of LCN2 in ALS

**Figure 3:**
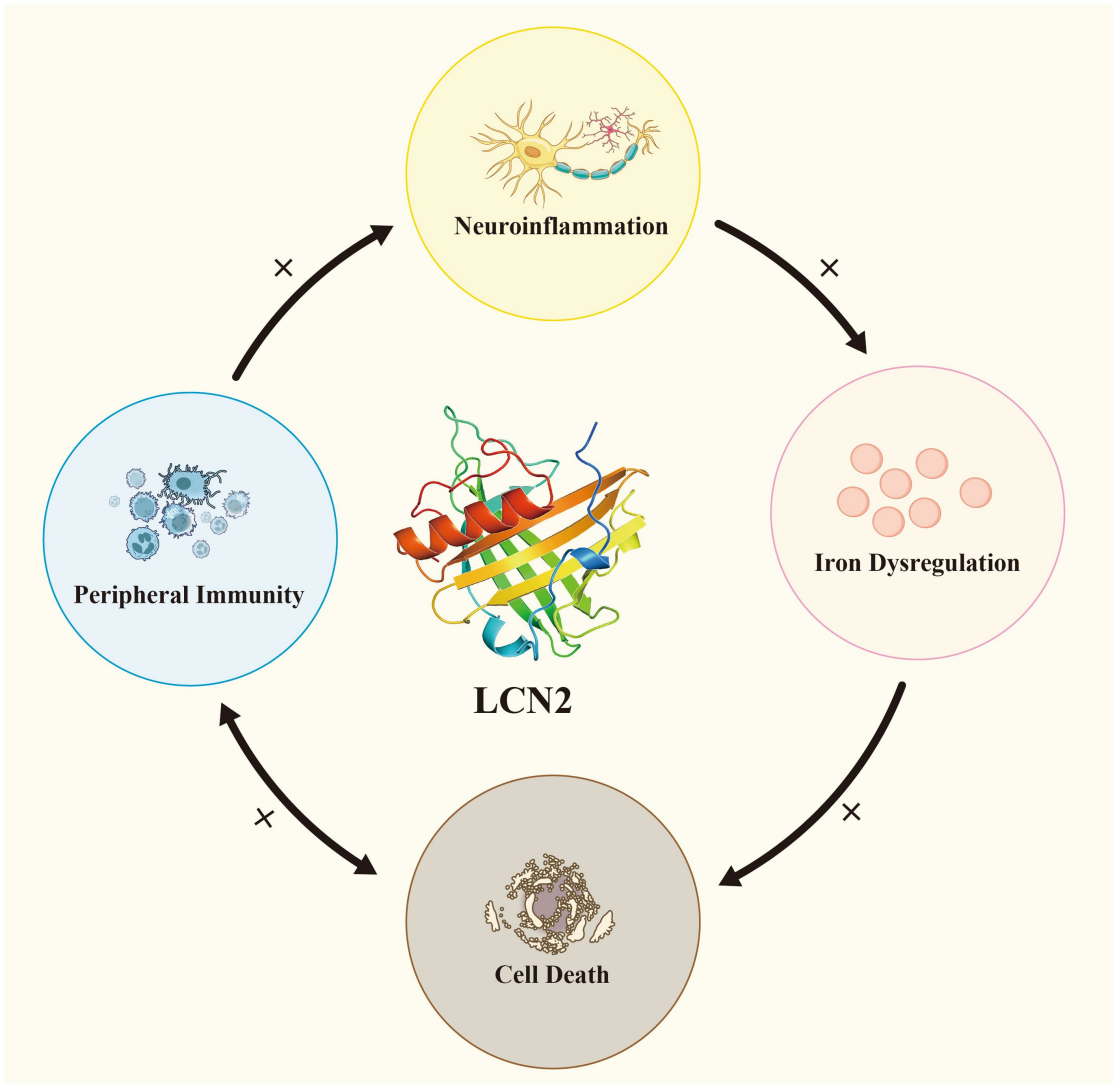
LCN2 establishes a complex central–peripheral linkage positive feedback loop in ALS. Neuroinflammation induces LCN2 upregulation, which in turn amplifies iron dysregulation and cell death. In the peripheral immune system, LCN2 can disrupt the blood–brain barrier and neuromuscular junctions, leading to neuronal damage and facilitating the entry of peripheral inflammation into the central nervous system. Neuronal death simultaneously activates glial cells and peripheral immune responses.

By integrating evidence from studies on neuroinflammation, iron dysregulation, cell death, and peripheral immunity, we propose a loop model of ALS pathogenesis centered on LCN2. In this model, LCN2 serves as a molecular bridge between the CNS and the peripheral immune system, amplifying pathological cascades through multiple positive feedback loops that accelerate disease progression. Neuroinflammation triggers LCN2 upregulation, which subsequently disrupts iron homeostasis and elevates ROS production, thereby driving protein aggregation and neuronal death. In the peripheral immune system, LCN2 exacerbates the disruption of NMJ and the BBB, facilitating the spread of peripheral inflammation into the CNS and leading to neuronal damage. Neuronal death, in turn, activates glial cells and peripheral immune responses, further enhancing LCN2 secretion and inflammatory signaling. This central-peripheral linkage mechanism provides a unified conceptual framework for ALS pathogenesis and suggests that LCN2 and its associated signaling pathways may serve as critical targets for future therapeutic interventions and biomarker development (Figure 3).

## 5 Clinical relevance of LCN2 in ALS

### 5.1 Biomarker potential

LCN2 has been recognized as a highly promising biomarker in recent years due to its convenient detection, high stability, and diverse sample sources (Chakraborty et al., 2012). Its detection samples include not only serum and plasma but also stable measurements in non-invasive samples such as urine and feces (Chassaing et al., 2012; Helanova et al., 2014), thereby enhancing patient compliance. Regarding detection techniques, conventional methods such as ELISA or colloidal gold assays enable rapid and sensitive testing. Notably, a rapid colloidal gold assay for LCN2 has entered clinical trials (national clinical trial number: NCT05450523), demonstrating feasibility for point-of-care diagnostics. Furthermore, LCN2 forms a complex with MMP-9, exhibiting protease-resistant properties (Janas et al., 2014). This stability during sample storage and processing provides a foundation for multicenter clinical studies. However, variations in sensitivity and quantitative standards exist across different detection methods, necessitating further validation of cross-platform consistency.

In neurodegenerative diseases, LCN2 has been recognized as a potential biomarker. For instance, AD patients exhibit elevated plasma LCN2 levels even during the mild cognitive impairment stage, and this elevation correlates with cerebrospinal fluid tau levels (Choi et al., 2011; Zhang et al., 2025; Doroszkiewicz et al., 2024). Cerebrospinal fluid LCN2 levels in vascular dementia patients exceed those in AD, suggesting its potential significance in differentiating between distinct pathological subtypes (Li X. et al., 2023). Furthermore, LCN2 levels rapidly increase in stroke and traumatic brain injury, correlating with early neurological deterioration (Kim et al., 2025; Laohavisudhi et al., 2024; Xie et al., 2023; Kim et al., 2023). As previously mentioned, plasma LCN2 levels are significantly elevated in ALS patients. Concurrently, postmortem analyses have revealed increased LCN2 expression in the motor cortex and spinal cord of ALS patients, further supporting its potential role as a diagnostic biomarker for ALS (Ngo et al., 2015; Petrozziello et al., 2020). However, current studies lack longitudinal assessments and have not incorporated precise stratification of ALS patients. Moreover, elevated LCN2 levels are not unique to ALS but are commonly observed across various neurological disorders.

As a biomarker, LCN2 has entered clinical trials for kidney disease, infectious diseases, and certain neurological disorders (Jung and Ryu, 2023; Fassett et al., 2011). Its ease of detection enables comprehensive disease assessment throughout the entire disease cycle. However, the future clinical application of LCN2 in ALS may still face several challenges, including the lack of standardized reference thresholds and internationally harmonized detection protocols for baseline LCN2 levels (McWilliam et al., 2014), the unclear characterization of LCN2 across different ALS subtypes and disease stages, and its limited pathological specificity, which necessitates integration with additional biomarkers to enhance diagnostic accuracy. Although no clinical trials targeting LCN2 in ALS have been conducted, these studies provide crucial evidence supporting its potential as a biomarker.

### 5.2 Advances and prospects in LCN2-targeted therapy

With growing understanding of the diverse pathogenic roles of LCN2 in ALS and other neurological diseases, therapies targeting LCN2 have attracted increasing attention. As a key molecule linking neuroinflammation, iron dysregulation, and cell death, modulating LCN2 or its related signaling pathways holds promise for the development of novel treatments for neurodegenerative diseases like ALS.

Current therapeutic approaches aim to regulate LCN2 expression and function at several levels, including gene transcription, post-transcriptional modification, protein processing, and interaction. At the transcriptional level, inflammatory stimuli activate the NF-κB pathway, thereby promoting LCN2 expression; conversely, proteasome inhibitors reduce LCN2 transcription by stabilizing IκB proteins (Chan et al., 2016). At the post-transcriptional level, specific microRNAs such as miRNA-130a and miRNA-138 directly target LCN2 messenger RNA (Larsen et al., 2014; Xiong et al., 2016). This regulatory approach exhibits high specificity, though its clinical translational feasibility remains unclear. At the post-translational level, regulation of the N-terminal signal peptide and associated pathways determines whether LCN2 is secreted extracellularly or degraded. Autophagy activators have demonstrated potential for reducing extracellular LCN2 levels (Goetz et al., 2000; Jung et al., 2023; Chan et al., 2016; Kjeldsen et al., 1993). Additionally, disrupting the LCN2-matrix metalloproteinase 9 (MMP9) complex has been proposed as a therapeutic strategy to directly block its pathological function (Song et al., 2014).

In recent years, various interventions targeting LCN2 have demonstrated efficacy in animal models of neurological disorders, spanning multiple levels from gene transcription regulation to pharmacological and non-pharmacological therapies. Key strategies include: small-molecule drugs and nucleic acid interventions that directly suppress LCN2 expression or function (Jung et al., 2023; Dekens et al., 2020; Deng et al., 2019; Braga et al., 2020; Zhang et al., 2019; Cui et al., 2022), targeted delivery platforms utilizing nanomedicine or cell-based carriers (Xiao et al., 2025; Vismara et al., 2020), neutralizing antibodies and biologics against LCN2 (Wang et al., 2020), and non-pharmacological physical therapies such as exercise, photobiomodulation, low-intensity pulsed ultrasound, and electroacupuncture (Wang et al., 2021; Yamaguchi et al., 2023; Sung et al., 2021; Chen et al., 2021). These studies validate the feasibility of intervening in LCN2 and its downstream signaling pathways from multiple perspectives, providing mechanistic evidence for its potential as a novel therapeutic target. However, numerous challenges remain in translating these findings from animal studies to clinical applications. First, LCN2 plays critical physiological roles in iron homeostasis and antimicrobial immunity; prolonged or systemic inhibition may lead to metabolic disorders and increased susceptibility to infections. Second, delivery barriers across the BBB, potential off-target effects, and the immunogenicity and long-term safety of nucleic acid therapies require careful evaluation. Additionally, some small molecules have demonstrated significant central nervous system side effects in humans. While nanodelivery may reduce systemic exposure, cross-species delivery efficiency and long-term toxicity remain unclear. To advance clinical translation, future research must address three key challenges: First, developing safe and efficient drug delivery strategies; second, establishing sensitive biomarker systems tailored to ALS’s high heterogeneity for precise patient stratification and efficacy assessment; third, exploring combined applications of LCN2 drug interventions with existing immunomodulatory, cell therapy, and antioxidant treatments to achieve multi-pathway, multidimensional comprehensive interventions. Regarding clinical priority, small-molecule drugs and neutralizing antibodies with established pharmacokinetic and safety profiles hold near-term translational potential. Nanodelivery platforms and autophagy-modulating strategies represent mid-to-long-term innovation directions. Non-pharmacological interventions like exercise and electroacupuncture, offering high safety and accessibility, may first enter clinical validation as adjunct or combination therapies. Overall, LCN2-targeted therapies hold promise but require cautious advancement. The optimal pathway involves gradually exploring clinical feasibility through small-scale, biomarker-driven early-phase trials grounded in robust preclinical research (Table 1).

**Table 1:** Therapeutic strategies targeting LCN2 in ALS: mechanisms, feasibility, safety, and translational potential.

Strategy category	Specific strategy (reference)	Mechanism	Feasibility	Safety	Clinical translation potential
Nucleic acid-based interventions	miR-138-5p exosome delivery (Deng et al., 2019)	Targeted inhibition of LCN2, reducing astrocyte inflammatory response and apoptosis	Dependent on exosome delivery system	Potential off-target effects, long-term safety not validated	Promising, but clinical delivery methods need optimization
	pRNA-RNAi LCN2 knockdown + iNSC transplantation (Braga et al., 2020)	RNAi suppresses LCN2 expression, improves scarring and inflammation, promotes neuronal survival	Synergistic effect when combined with stem cell transplantation	Risk of long-term RNAi silencing and immune responses	Requires further long-term validation, combination strategy has potential
Nanoparticle-based delivery	AAV-shRNA targeting LCN2 (Xiao et al., 2025)	Downregulates LCN2 and promotes A2 astrocyte conversion	AAV and nanoparticle platforms are under development	Risks related to gene therapy immune response and toxicity	Cutting-edge approach, translation potential is considerable
	Rolipram nanoparticle delivery (Vismara et al., 2020)	Suppresses A1 astrocyte inflammation, reduces LCN2 expression	Drug known, delivery via carrier improves specificity	Rolipram has intrinsic side effects, dosage control needed	Optimization of delivery could enable translation
Small molecules and chemical modulators	Sailuotong capsule (Zhang et al., 2019)	Reduces astrocyte proliferation, lowers LCN2 expression, inhibits LCN2-JAK2/STAT3 signaling	Naturally derived, partially applied clinically	Relatively low toxicity	Favorable translational potential, may serve as adjuvant therapy
	Iron chelators Deferoxamine/Deferiprone (Dekens et al., 2020; Cui et al., 2022)	Reduces iron load, induces autophagy, suppresses LCN2 upregulation	Well-established drugs, high feasibility	Long-term iron deficiency risk, requires monitoring	Established clinical use, high translation potential
	Proteasome inhibitors and autophagy activators (Rapamycin) (Jung et al., 2023)	NF-κB inhibition or autophagy enhancement reduces LCN2	Mechanistically clear, drugs are non-specific	Proteasome toxicity, autophagy modulation requires balance	Needs specificity optimization, short-term translation limited
Immunobiologics	LCN2 monoclonal antibody (Wang et al., 2020)	Neutralizes LCN2	Strong effects in animal models	Risk of immune-related side effects	If optimized, high breakthrough potential
Physical interventions	Exercise rehabilitation (Yamaguchi et al., 2023)	Modifies astrocyte lineage, reduces number of LCN2-expressing astrocytes	Highly feasible, easy to implement	High safety	Clinically feasible as rehabilitation adjunct, limited efficacy
	Photobiomodulation (Wang et al., 2021)	Reduces LCN2 expression, inhibits LCN2-JAK2/STAT3 signaling	Validated in animal studies, simple to operate	Relatively high safety	Can serve as rehabilitation adjunct, clinical studies feasible
	Low-intensity pulsed ultrasound (Sung et al., 2021)	Enhances GDNF, inhibits LCN2-mediated inflammation	Non-invasive, operable	Low potential tissue heating risk	Can be combined with existing interventions, moderate translation potential
	Electroacupuncture (Chen et al., 2021)	Suppresses LCN2 upregulation, improves astrocyte activation	Mature technique, already clinically applied	High safety, minimal side effects	Can quickly enter clinical trials

AAV, adeno-associated virus; ALS, amyotrophic lateral sclerosis; CNS, central nervous system; GDNF, glial cell line-derived neurotrophic factor; iNSC, induced neural stem cell; JAK2, janus kinase 2; LCN2, lipocalin 2; miR, microRNA; NF-κB, nuclear factor kappa-light-chain-enhancer of activated B cells; RNAi, RNA interference; ROS, reactive oxygen species; shRNA, short hairpin RNA; STAT3, signal transducer and activator of transcription 3.

In summary, interventions targeting LCN2 have demonstrated promising efficacy in preclinical models. These interventions encompass pharmacological, genetic, and non-pharmacological approaches. Despite these advances, clinical translation remains constrained by safety concerns, delivery challenges, and the influence of LCN2’s own physiological functions. Future efforts should focus on meticulously designed, biomarker-guided early-phase clinical trials that integrate multimodal strategies. Such approaches may establish LCN2 as a novel therapeutic target for ALS and other neurodegenerative diseases.

### 5.3 Further considerations: LCN2 in ALS at the intersection of immune regulation and cardiovascular function

Beyond strategies directly targeting LCN2, the regulation of the immune system and cardiovascular function also plays a crucial role in the progression of neurodegenerative diseases such as ALS. Recent studies have provided important clues for understanding the role of immunity in the progression of neurodegenerative diseases. In AD patients, a decreased proportion of peripheral blood T lymphocytes is closely associated with poorer neurological function scores (Willman et al., 2024), suggesting that T cell immune homeostasis may exert neuroprotective effects in the early stages of the disease. In ALS, T cells constitute the predominant lymphocyte subtype infiltrating the central nervous system, with their infiltration closely associated with adaptive immune responses in regions of neuronal damage (Tuddenham et al., 2025). Studies show that CD4^+^ T cell accumulation is often accompanied by elevated CCL2 and activated microglia, suggesting T cells may play a central role in disease onset and progression. Concurrently, clinical evidence indicates that increased effector T cell ratios correlate with poor prognosis and rapid disease progression, whereas enhanced regulatory T cells are associated with better survival and slower functional decline (Yazdani et al., 2022). Collectively, these findings suggest a double-edged sword role for T cells in ALS. Integrating the functional characteristics of LCN2 explored in this study, its upregulation may indirectly disrupt T cell immune homeostasis by promoting neutrophil recruitment and activating astrocytes and microglia, thereby forming a pro-inflammatory–neurotoxicity positive feedback loop. This mechanism offers significant implications for disease management: beyond direct targeting of LCN2, future interventions targeting immune responses hold potential. Specifically, cell therapies or immunomodulators targeting specific T cell subsets may represent promising avenues for slowing ALS progression.

Increasing evidence suggests that ALS progression is influenced not only by intrinsic neuronal mechanisms but may also be closely linked to cardiovascular function. Our team’s previous Mendelian randomization study revealed that elevated systolic blood pressure increases ALS risk, while elevated diastolic blood pressure may exert a protective effect, indicating the potential significance of hemodynamic patterns in ALS pathogenesis (Xia et al., 2022). Similar to the vascular-neuroinflammatory mechanism proposed in studies of gestational hypertension and postpartum depression (Kalawatia et al., 2025), abnormal blood pressure fluctuations in ALS may accelerate neurodegenerative processes through upregulation of pro-inflammatory factors, oxidative stress, and metabolic imbalance. As a key molecule in inflammation and iron homeostasis, LCN2 is not only closely associated with neuroinflammation but also plays a significant role in cardiovascular events such as atherosclerosis and myocardial injury (Liu et al., 2025). This cross-regulatory mechanism suggests that modulating cardiovascular function may partially alleviate LCN2-associated neurotoxicity. To optimize ALS treatment outcomes, future approaches could integrate hemodynamic interventions with existing pharmacotherapies. Studies have reported that regular exercise training and electroacupuncture therapy can improve blood pressure variability (Carlson et al., 2014; Longhurst and Tjen, 2017). As mentioned earlier, these approaches can downregulate inflammation levels and reduce LCN2 expression (Yamaguchi et al., 2023; Chen et al., 2021). These interventions may offer low-risk, highly feasible adjunctive treatment strategies for clinical practice.

## 6 Conclusion and future directions

LCN2, as a multifunctional inflammatory mediator, plays a critical role in the pathogenesis and progression of ALS. Its expression is significantly upregulated in both the central nervous system and peripheral blood, mainly through astrocytes and neutrophils. LCN2 contributes to neuroinflammation and pathological microenvironmental changes around motor neurons. Its expression is regulated by several signaling pathways, including NF-κB, C/EBP, STAT3, and PERK-eIF2*α*-ATF4. Functionally, LCN2 is implicated in multiple mechanisms including neuroinflammation, iron homeostasis disruption, cell death, and peripheral immunity. In this review, we propose a central-peripheral linkage mechanism model centered on LCN2, which integrates these four key pathways and emphasizes their mutual amplification through positive feedback loops, thereby accelerating ALS disease progression. Clinically, LCN2 holds significant potential as a biomarker due to its ease of detection, high stability, and accessibility from diverse biological samples. However, challenges remain, including variations in baseline levels across populations, insufficient disease specificity, and a lack of standardization across different detection platforms. Similarly, therapeutic strategies targeting LCN2—encompassing small-molecule inhibitors, nucleic acid interventions, neutralizing antibodies, nanodelivery platforms, and non-pharmacological approaches—have demonstrated efficacy in preclinical models. However, their translation into clinical practice remains hindered by LCN2’s physiological roles in iron metabolism and host defense, potential off-target effects, and challenges in central nervous system delivery. Furthermore, LCN2-mediated neurotoxicity may be linked to immune regulation and cardiovascular function, suggesting that integrated strategies combining LCN2-targeted therapy, immunomodulation, and hemodynamic interventions could potentially optimize patient outcomes. Based on this, LCN2 represents both a pathogenic amplifier and a promising therapeutic and biomarker target, warranting further investigation to translate mechanistic insights into clinical applications in ALS.

**Figure 4:**
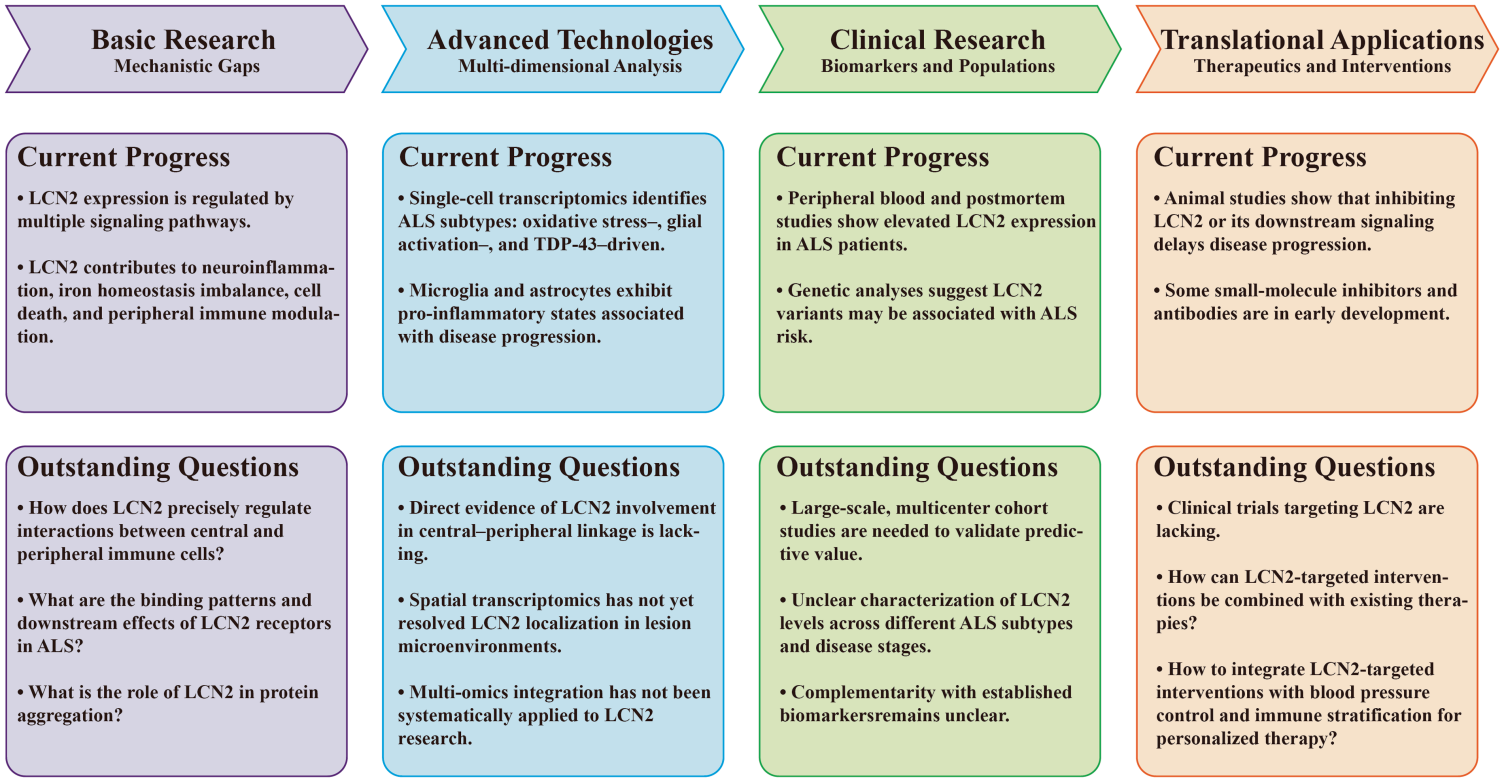
Roles and translational potential of LCN2 in ALS. The roadmap is divided into four sections: basic research: mechanistic gaps, advanced technologies: multi-dimensional analysis, clinical research: biomarkers and populations, and translational applications: therapeutics and interventions. Each section highlights both current progress and outstanding questions to delineate the evolving understanding and future directions of LCN2 research in ALS. ALS, amyotrophic lateral sclerosis; CNS, central nervous system; GFAP, glial fibrillary acidic protein; LCN2, lipocalin-2; NfL, neurofilament light chain; TDP-43, TAR DNA-binding Protein 43.

In the future, although LCN2 has been demonstrated to exert multiple pathogenic effects in ALS and other neurodegenerative diseases, its specific molecular mechanisms remain largely unknown. For instance, the roles of LCN2 in neuroinflammation, iron homeostasis disruption, cell death, and peripheral immune regulation lack systematic validation. Critical questions remain regarding how LCN2 precisely regulates central–peripheral immune crosstalk, the binding patterns and downstream effects of its receptors in ALS, and its potential role in protein aggregation. Moreover, spatial transcriptomics has yet to clarify LCN2 localization within lesion microenvironments, and multi-omics integration has not been systematically applied to elucidate its network-level mechanisms. Concurrently, clinical evidence remains limited: large-scale, multicenter cohort studies are lacking. The complementarity of LCN2 with established biomarkers such as neurofilament light chain and glial fibrillary acidic protein remains unclear, and standardized data on baseline levels and dynamic changes across populations are insufficient. From a translational perspective, clinical trials specifically targeting LCN2 are still absent. Questions persist regarding how to effectively combine LCN2-targeted interventions with existing therapies, blood pressure control, and immune stratification to achieve personalized treatment. Future research should prioritize multi-omics integration analysis, combining single-cell sequencing, proteomics, and metabolomics to comprehensively decipher LCN2-mediated molecular networks. Leveraging data-driven systems biology models, it is essential to explore its key pathways in central–peripheral linkage, offering new perspectives on understanding ALS progression mechanisms. Clinically, LCN2 levels should be incorporated into patient stratification and treatment response prediction to guide precision medicine and personalized interventions. Integrating multi-omics data with clinical evidence will help bridge existing research gaps and lay a solid foundation for the clinical translation of LCN2-targeted therapies (Figure 4).

## Glossary

AD: Alzheimer’s disease
ALS: Amyotrophic lateral sclerosis
ATF4: activating transcription factor 4
BBB: blood–brain barrier
C/EBP: CCAAT/enhancer-binding protein
eIF2α: eukaryotic initiation factor 2α
FUS: fused in sarcoma
GFAP: glial fibrillary acidic protein
IL: interleukin
JAK: Janus kinase
MMP9: matrix metalloproteinase 9
MS: multiple sclerosis
NMJ: neuromuscular junctions
NO: nitric oxide
NF-κB: nuclear factor-κB
PD: Parkinson’s disease
PERK: Protein Kinase RNA-like Endoplasmic Reticulum Kinase
ROS: reactive oxygen species
SOD1: superoxide dismutase 1
STAT3: signal transducer and activator of transcription 3
TDP-43: TAR DNA-binding protein 43
TNF-α: tumor necrosis factor-α
TRPC: transient receptor potential canonical

## References

[ref1] Abella V. Scotece M. Conde J. Gómez R. Lois A. Pino J.. (2015). The potential of lipocalin-2/NGAL as biomarker for inflammatory and metabolic diseases. Biomarkers 20, 565–571. doi: 10.3109/1354750x.2015.1123354, PMID: 26671823 PMC4819811

[ref2] Allen N. J. Lyons D. A. (2018). Glia as architects of central nervous system formation and function. Science 362, 181–185. doi: 10.1126/science.aat0473, PMID: 30309945 PMC6292669

[ref3] Amersfoort J. Schaftenaar F. H. Douna H. van Santbrink P. J. Kröner M. J. van Puijvelde G. H. M.. (2018). Lipocalin-2 contributes to experimental atherosclerosis in a stage-dependent manner. Atherosclerosis 275, 214–224. doi: 10.1016/j.atherosclerosis.2018.06.015, PMID: 29960897

[ref4] Bachman M. A. Lenio S. Schmidt L. Oyler J. E. Weiser J. N. (2012). Interaction of lipocalin 2, transferrin, and siderophores determines the replicative niche of *Klebsiella pneumoniae* during pneumonia. mBio 3:e00224-11. doi: 10.1128/mBio.00224-11, PMID: 23169997 PMC3509427

[ref5] Bao G. H. Barasch J. Xu J. Wang W. Hu F. L. Deng S. X. (2015a). Purification and structural characterization of "simple catechol", the NGAL-Siderocalin siderophore in human urine. RSC Adv. 5, 28527–28535. doi: 10.1039/c5ra02509e, PMID: 26257890 PMC4527557

[ref6] Bao G. Clifton M. Hoette T. M. Mori K. Deng S. X. Qiu A.. (2010). Iron traffics in circulation bound to a siderocalin (Ngal)-catechol complex. Nat. Chem. Biol. 6, 602–609. doi: 10.1038/nchembio.402, PMID: 20581821 PMC2907470

[ref7] Bao G. H. Ho C. T. Barasch J. (2015b). The ligands of neutrophil gelatinase-associated lipocalin. RSC Adv. 5, 104363–104374. doi: 10.1039/c5ra18736b, PMID: 27617081 PMC5014392

[ref8] Bao G. H. Xu J. Hu F. L. Wan X. C. Deng S. X. Barasch J. (2013). EGCG inhibit chemical reactivity of iron through forming an Ngal-EGCG-iron complex. Biometals 26, 1041–1050. doi: 10.1007/s10534-013-9681-8, PMID: 24158698 PMC4416964

[ref9] Beard J. L. Connor J. R. (2003). Iron status and neural functioning. Annu. Rev. Nutr. 23, 41–58. doi: 10.1146/annurev.nutr.23.020102.075739, PMID: 12704220

[ref10] Beers D. R. Appel S. H. (2019). Immune dysregulation in amyotrophic lateral sclerosis: mechanisms and emerging therapies. Lancet Neurol. 18, 211–220. doi: 10.1016/s1474-4422(18)30394-6, PMID: 30663610

[ref11] Berard J. L. Zarruk J. G. Arbour N. Prat A. Yong V. W. Jacques F. H.. (2012). Lipocalin 2 is a novel immune mediator of experimental autoimmune encephalomyelitis pathogenesis and is modulated in multiple sclerosis. Glia 60, 1145–1159. doi: 10.1002/glia.22342, PMID: 22499213

[ref12] Bi F. Huang C. Tong J. Qiu G. Huang B. Wu Q.. (2013). Reactive astrocytes secrete lcn2 to promote neuron death. Proc. Natl. Acad. Sci. USA 110, 4069–4074. doi: 10.1073/pnas.1218497110, PMID: 23431168 PMC3593910

[ref13] Borregaard N. Cowland J. B. (2006). Neutrophil gelatinase-associated lipocalin, a siderophore-binding eukaryotic protein. Biometals 19, 211–215. doi: 10.1007/s10534-005-3251-7, PMID: 16718606

[ref14] Braga A. Bandiera S. Verheyen J. Hamel R. Rutigliani C. Edenhofer F.. (2020). Combination of in situ Lcn2 pRNA-RNAi nanotherapeutics and iNSC transplantation ameliorates experimental SCI in mice. Mol. Ther. 28, 2677–2690. doi: 10.1016/j.ymthe.2020.08.001, PMID: 32877696 PMC7704756

[ref15] Cady J. Allred P. Bali T. Pestronk A. Goate A. Miller T. M.. (2015). Amyotrophic lateral sclerosis onset is influenced by the burden of rare variants in known amyotrophic lateral sclerosis genes. Ann. Neurol. 77, 100–113. doi: 10.1002/ana.24306, PMID: 25382069 PMC4293318

[ref16] Cakal E. Ozkaya M. Engin-Ustun Y. Ustun Y. (2011). Serum lipocalin-2 as an insulin resistance marker in patients with polycystic ovary syndrome. J. Endocrinol. Investig. 34, 97–100. doi: 10.1007/bf03347037, PMID: 20511727

[ref17] Carlson D. J. Dieberg G. Hess N. C. Millar P. J. Smart N. A. (2014). Isometric exercise training for blood pressure management: a systematic review and meta-analysis. Mayo Clin. Proc. 89, 327–334. doi: 10.1016/j.mayocp.2013.10.030, PMID: 24582191

[ref18] Chakraborty S. Kaur S. Guha S. Batra S. K. (2012). The multifaceted roles of neutrophil gelatinase associated lipocalin (NGAL) in inflammation and cancer. Biochim. Biophys. Acta 1826, 129–169. doi: 10.1016/j.bbcan.2012.03.008, PMID: 22513004 PMC3362670

[ref19] Chan Y. K. Sung H. K. Jahng J. W. Kim G. H. Han M. Sweeney G. (2016). Lipocalin-2 inhibits autophagy and induces insulin resistance in H9c2 cells. Mol. Cell. Endocrinol. 430, 68–76. doi: 10.1016/j.mce.2016.04.006, PMID: 27090568

[ref20] Chassaing B. Srinivasan G. Delgado M. A. Young A. N. Gewirtz A. T. Vijay-Kumar M. (2012). Fecal lipocalin 2, a sensitive and broadly dynamic non-invasive biomarker for intestinal inflammation. PLoS One 7:e44328. doi: 10.1371/journal.pone.0044328, PMID: 22957064 PMC3434182

[ref21] Chen Y. H. Xie S. Y. Chen C. W. Lu D. Y. (2021). Electroacupuncture improves repeated social defeat stress-elicited social avoidance and anxiety-like behaviors by reducing Lipocalin-2 in the hippocampus. Mol. Brain 14:150. doi: 10.1186/s13041-021-00860-0, PMID: 34565419 PMC8474847

[ref22] Chia R. Chiò A. Traynor B. J. (2018). Novel genes associated with amyotrophic lateral sclerosis: diagnostic and clinical implications. Lancet Neurol. 17, 94–102. doi: 10.1016/s1474-4422(17)30401-5, PMID: 29154141 PMC5901717

[ref23] Chia W. J. Dawe G. S. Ong W. Y. (2011). Expression and localization of the iron-siderophore binding protein lipocalin 2 in the normal rat brain and after kainate-induced excitotoxicity. Neurochem. Int. 59, 591–599. doi: 10.1016/j.neuint.2011.04.007, PMID: 21683107

[ref24] Chia W. J. Tan F. C. Ong W. Y. Dawe G. S. (2015). Expression and localisation of brain-type organic cation transporter (BOCT/24p3R/LCN2R) in the normal rat hippocampus and after kainate-induced excitotoxicity. Neurochem. Int. 87, 43–59. doi: 10.1016/j.neuint.2015.04.009, PMID: 26004810

[ref25] Choi J. Lee H. W. Suk K. (2011). Increased plasma levels of lipocalin 2 in mild cognitive impairment. J. Neurol. Sci. 305, 28–33. doi: 10.1016/j.jns.2011.03.023, PMID: 21463871

[ref26] Cohen T. J. Hwang A. W. Unger T. Trojanowski J. Q. Lee V. M. (2012). Redox signalling directly regulates TDP-43 via cysteine oxidation and disulphide cross-linking. EMBO J. 31, 1241–1252. doi: 10.1038/emboj.2011.471, PMID: 22193716 PMC3297986

[ref27] Cowland J. B. Borregaard N. (1997). Molecular characterization and pattern of tissue expression of the gene for neutrophil gelatinase-associated lipocalin from humans. Genomics 45, 17–23. doi: 10.1006/geno.1997.4896, PMID: 9339356

[ref28] Cui J. Yuan Y. Wang J. Song N. Xie J. (2022). Desferrioxamine ameliorates lipopolysaccharide-induced Lipocalin-2 upregulation via autophagy activation in primary astrocytes. Mol. Neurobiol. 59, 2052–2067. doi: 10.1007/s12035-021-02687-1, PMID: 35040039

[ref29] Dadon-Nachum M. Melamed E. Offen D. (2011). The "dying-back" phenomenon of motor neurons in ALS. J. Mol. Neurosci. 43, 470–477. doi: 10.1007/s12031-010-9467-1 21057983

[ref30] Dekens D. W. De Deyn P. P. Sap F. Eisel U. L. M. Naudé P. J. W. (2020). Iron chelators inhibit amyloid-β-induced production of lipocalin 2 in cultured astrocytes. Neurochem. Int. 132:104607. doi: 10.1016/j.neuint.2019.104607, PMID: 31760034

[ref31] Dekens D. W. Eisel U. L. M. Gouweleeuw L. Schoemaker R. G. De Deyn P. P. Naudé P. J. W. (2021). Lipocalin 2 as a link between ageing, risk factor conditions and age-related brain diseases. Ageing Res. Rev. 70:101414. doi: 10.1016/j.arr.2021.101414, PMID: 34325073

[ref32] Dekens D. W. Naudé P. J. W. Keijser J. N. Boerema A. S. De Deyn P. P. Eisel U. L. M. (2018). Lipocalin 2 contributes to brain iron dysregulation but does not affect cognition, plaque load, and glial activation in the J20 Alzheimer mouse model. J. Neuroinflammation 15:330. doi: 10.1186/s12974-018-1372-5, PMID: 30501637 PMC6267886

[ref33] Deng Y. Chen D. Gao F. Lv H. Zhang G. Sun X.. (2019). Exosomes derived from microRNA-138-5p-overexpressing bone marrow-derived mesenchymal stem cells confer neuroprotection to astrocytes following ischemic stroke via inhibition of LCN2. J. Biol. Eng. 13:71. doi: 10.1186/s13036-019-0193-0, PMID: 31485266 PMC6714399

[ref34] Devarajan P. (2007). Neutrophil gelatinase-associated lipocalin: new paths for an old shuttle. Cancer Ther. 5, 463–470, PMID: 18449360 PMC2361391

[ref35] Devireddy L. R. Gazin C. Zhu X. Green M. R. (2005). A cell-surface receptor for lipocalin 24p3 selectively mediates apoptosis and iron uptake. Cell 123, 1293–1305. doi: 10.1016/j.cell.2005.10.027, PMID: 16377569

[ref36] Dizin E. Hasler U. Nlandu-Khodo S. Fila M. Roth I. Ernandez T.. (2013). Albuminuria induces a proinflammatory and profibrotic response in cortical collecting ducts via the 24p3 receptor. Am. J. Physiol. Renal Physiol. 305, F1053–F1063. doi: 10.1152/ajprenal.00006.2013, PMID: 23884139

[ref37] Doroszkiewicz J. Kulczyńska-Przybik A. Dulewicz M. Mroczko J. Borawska R. Słowik A.. (2024). Associations between microglia and astrocytic proteins and tau biomarkers across the continuum of Alzheimer's disease. Int. J. Mol. Sci. 25:7543. doi: 10.3390/ijms25147543, PMID: 39062786 PMC11277045

[ref38] Dzhashiashvili Y. Monckton C. P. Shah H. S. Kunjamma R. B. Popko B. (2019). The UPR-PERK pathway is not a promising therapeutic target for mutant SOD1-induced ALS. Neurobiol. Dis. 127, 527–544. doi: 10.1016/j.nbd.2019.03.024, PMID: 30923003 PMC6588429

[ref39] Eidson L. N. Kannarkat G. T. Barnum C. J. Chang J. Chung J. Caspell-Garcia C.. (2017). Candidate inflammatory biomarkers display unique relationships with alpha-synuclein and correlate with measures of disease severity in subjects with Parkinson's disease. J. Neuroinflammation 14:164. doi: 10.1186/s12974-017-0935-1, PMID: 28821274 PMC5563061

[ref40] Fan Y. Li X. Ma J. Yang D. Liang K. Shen Y.. (2024). Increased plasma lipocalin-2 levels are associated with nonmotor symptoms and neuroimaging features in patients with Parkinson's disease. J. Neurosci. Res. 102:e25303. doi: 10.1002/jnr.25303, PMID: 38361408

[ref41] Fang W. K. Xu L. Y. Lu X. F. Liao L. D. Cai W. J. Shen Z. Y.. (2007). A novel alternative spliced variant of neutrophil gelatinase-associated lipocalin receptor in oesophageal carcinoma cells. Biochem. J. 403, 297–303. doi: 10.1042/bj20060836, PMID: 17253959 PMC1874233

[ref42] Fassett R. G. Venuthurupalli S. K. Gobe G. C. Coombes J. S. Cooper M. A. Hoy W. E. (2011). Biomarkers in chronic kidney disease: a review. Kidney Int. 80, 806–821. doi: 10.1038/ki.2011.198, PMID: 21697815

[ref43] Feldman E. L. Goutman S. A. Petri S. Mazzini L. Savelieff M. G. Shaw P. J.. (2022). Amyotrophic lateral sclerosis. Lancet 400, 1363–1380. doi: 10.1016/s0140-6736(22)01272-7, PMID: 36116464 PMC10089700

[ref44] Ferreira A. C. Dá Mesquita S. Sousa J. C. Correia-Neves M. Sousa N. Palha J. A.. (2015). From the periphery to the brain: Lipocalin-2, a friend or foe? Prog. Neurobiol. 131, 120–136. doi: 10.1016/j.pneurobio.2015.06.005, PMID: 26159707

[ref45] Ferreira A. C. Santos T. Sampaio-Marques B. Novais A. Mesquita S. D. Ludovico P.. (2018). Lipocalin-2 regulates adult neurogenesis and contextual discriminative behaviours. Mol. Psychiatry 23, 1031–1039. doi: 10.1038/mp.2017.95, PMID: 28485407

[ref46] Flo T. H. Smith K. D. Sato S. Rodriguez D. J. Holmes M. A. Strong R. K.. (2004). Lipocalin 2 mediates an innate immune response to bacterial infection by sequestrating iron. Nature 432, 917–921. doi: 10.1038/nature03104, PMID: 15531878

[ref47] Flower D. R. (1994). The lipocalin protein family: a role in cell regulation. FEBS Lett. 354, 7–11. doi: 10.1016/0014-5793(94)01078-1, PMID: 7957904

[ref48] Flower D. R. (1996). The lipocalin protein family: structure and function. Biochem. J. 318, 1–14. doi: 10.1042/bj3180001, PMID: 8761444 PMC1217580

[ref49] Flower D. R. (2000). Experimentally determined lipocalin structures. Biochim. Biophys. Acta 1482, 46–56. doi: 10.1016/s0167-4838(00)00147-3, PMID: 11058746

[ref50] Flower D. R. North A. C. Sansom C. E. (2000). The lipocalin protein family: structural and sequence overview. Biochim. Biophys. Acta 1482, 9–24. doi: 10.1016/s0167-4838(00)00148-5 11058743

[ref51] Gaudet A. D. Fonken L. K. (2018). Glial cells shape pathology and repair after spinal cord injury. Neurotherapeutics 15, 554–577. doi: 10.1007/s13311-018-0630-7, PMID: 29728852 PMC6095774

[ref52] Gidday J. M. Gasche Y. G. Copin J. C. Shah A. R. Perez R. S. Shapiro S. D.. (2005). Leukocyte-derived matrix metalloproteinase-9 mediates blood-brain barrier breakdown and is proinflammatory after transient focal cerebral ischemia. Am. J. Physiol. Heart Circ. Physiol. 289, H558–H568. doi: 10.1152/ajpheart.01275.2004, PMID: 15764676

[ref53] Goetz D. H. Holmes M. A. Borregaard N. Bluhm M. E. Raymond K. N. Strong R. K. (2002). The neutrophil lipocalin NGAL is a bacteriostatic agent that interferes with siderophore-mediated iron acquisition. Mol. Cell 10, 1033–1043. doi: 10.1016/s1097-2765(02)00708-6 12453412

[ref54] Goetz D. H. Willie S. T. Armen R. S. Bratt T. Borregaard N. Strong R. K. (2000). Ligand preference inferred from the structure of neutrophil gelatinase associated lipocalin. Biochemistry 39, 1935–1941. doi: 10.1021/bi992215v, PMID: 10684642

[ref55] Goutman S. A. Chen K. S. Paez-Colasante X. Feldman E. L. (2018). Emerging understanding of the genotype-phenotype relationship in amyotrophic lateral sclerosis. Handb. Clin. Neurol. 148, 603–623. doi: 10.1016/b978-0-444-64076-5.00039-9, PMID: 29478603

[ref56] Greenhalgh A. D. David S. Bennett F. C. (2020). Immune cell regulation of glia during CNS injury and disease. Nat. Rev. Neurosci. 21, 139–152. doi: 10.1038/s41583-020-0263-9, PMID: 32042145

[ref57] Guo C. Steinberg L. K. Cheng M. Song J. H. Henderson J. P. Gross M. L. (2020). Site-specific Siderocalin binding to ferric and ferric-free Enterobactin as revealed by mass spectrometry. ACS Chem. Biol. 15, 1154–1160. doi: 10.1021/acschembio.9b00741, PMID: 31869199 PMC7236765

[ref58] Hare D. Ayton S. Bush A. Lei P. (2013). A delicate balance: Iron metabolism and diseases of the brain. Front. Aging Neurosci. 5:34. doi: 10.3389/fnagi.2013.00034, PMID: 23874300 PMC3715022

[ref59] He X. Zhang L. Yao X. Hu J. Yu L. Jia H.. (2013). Association studies of MMP-9 in Parkinson's disease and amyotrophic lateral sclerosis. PLoS One 8:e73777. doi: 10.1371/journal.pone.0073777, PMID: 24040066 PMC3767588

[ref60] Helanova K. Spinar J. Parenica J. (2014). Diagnostic and prognostic utility of neutrophil gelatinase-associated lipocalin (NGAL) in patients with cardiovascular diseases--review. Kidney Blood Press. Res. 39, 623–629. doi: 10.1159/000368474 25531230

[ref61] Hochmeister S. Engel O. Adzemovic M. Z. Pekar T. Kendlbacher P. Zeitelhofer M.. (2016). Lipocalin-2 as an infection-related biomarker to predict clinical outcome in ischemic stroke. PLoS One 11:e0154797. doi: 10.1371/journal.pone.0154797, PMID: 27152948 PMC4859492

[ref62] Hovens I. B. van Leeuwen B. L. Mariani M. A. Kraneveld A. D. Schoemaker R. G. (2016). Postoperative cognitive dysfunction and neuroinflammation; cardiac surgery and abdominal surgery are not the same. Brain Behav. Immun. 54, 178–193. doi: 10.1016/j.bbi.2016.02.003, PMID: 26867718

[ref63] Huang C. Huang B. Bi F. Yan L. H. Tong J. Huang J.. (2014). Profiling the genes affected by pathogenic TDP-43 in astrocytes. J. Neurochem. 129, 932–939. doi: 10.1111/jnc.12660, PMID: 24447103 PMC4066372

[ref64] Hvidberg V. Jacobsen C. Strong R. K. Cowland J. B. Moestrup S. K. Borregaard N. (2005). The endocytic receptor megalin binds the iron transporting neutrophil-gelatinase-associated lipocalin with high affinity and mediates its cellular uptake. FEBS Lett. 579, 773–777. doi: 10.1016/j.febslet.2004.12.031, PMID: 15670845

[ref65] Ilieva H. Vullaganti M. Kwan J. (2023). Advances in molecular pathology, diagnosis, and treatment of amyotrophic lateral sclerosis. BMJ 383:e075037. doi: 10.1136/bmj-2023-075037, PMID: 37890889 PMC10603569

[ref66] Jaberi S. A. Cohen A. D'Souza C. Abdulrazzaq Y. M. Ojha S. Bastaki S.. (2021). Lipocalin-2: structure, function, distribution and role in metabolic disorders. Biomed. Pharmacother. 142:112002. doi: 10.1016/j.biopha.2021.112002, PMID: 34463264

[ref67] Janas R. M. Ochocińska A. Snitko R. Dudka D. Kierkuś J. Teisseyre M.. (2014). Neutrophil gelatinase-associated lipocalin in blood in children with inflammatory bowel disease. J. Gastroenterol. Hepatol. 29, 1883–1889. doi: 10.1111/jgh.12597, PMID: 24720485

[ref68] Jha M. K. Lee S. Park D. H. Kook H. Park K. G. Lee I. K.. (2015). Diverse functional roles of lipocalin-2 in the central nervous system. Neurosci. Biobehav. Rev. 49, 135–156. doi: 10.1016/j.neubiorev.2014.12.006, PMID: 25511817

[ref69] Jiang Q. Wei Q. Zhang L. Yang T. Lin J. Xiao Y.. (2024). Peripheral immunity relate to disease progression and prognosis in amyotrophic lateral sclerosis. Amyotroph. Lateral Scler. Frontotemporal Degener. 25, 465–474. doi: 10.1080/21678421.2024.2306969, PMID: 38270154

[ref70] Joppe K. Roser A. E. Maass F. Lingor P. (2019). The contribution of Iron to protein aggregation disorders in the central nervous system. Front. Neurosci. 13:15. doi: 10.3389/fnins.2019.00015, PMID: 30723395 PMC6350163

[ref71] Jung B. K. Park Y. Yoon B. Bae J. S. Han S. W. Heo J. E.. (2023). Reduced secretion of LCN2 (lipocalin 2) from reactive astrocytes through autophagic and proteasomal regulation alleviates inflammatory stress and neuronal damage. Autophagy 19, 2296–2317. doi: 10.1080/15548627.2023.2180202, PMID: 36781380 PMC10351455

[ref72] Jung B. K. Ryu K. Y. (2023). Lipocalin-2: a therapeutic target to overcome neurodegenerative diseases by regulating reactive astrogliosis. Exp. Mol. Med. 55, 2138–2146. doi: 10.1038/s12276-023-01098-7, PMID: 37779143 PMC10618504

[ref73] Kalawatia M. Lucke-Wold B. Mehrunkar A. (2025). Closer look at the cardiovascular and metabolic predictors of postpartum depression. World J. Psychiatry 15:106283. doi: 10.5498/wjp.v15.i6.106283, PMID: 40574759 PMC12188881

[ref74] Kang S. S. Ren Y. Liu C. C. Kurti A. Baker K. E. Bu G.. (2018). Lipocalin-2 protects the brain during inflammatory conditions. Mol. Psychiatry 23, 344–350. doi: 10.1038/mp.2016.243, PMID: 28070126 PMC5503822

[ref75] Kang H. Shin H. J. An H. S. Jin Z. Lee J. Y. Lee J.. (2021). Role of Lipocalin-2 in amyloid-Beta oligomer-induced mouse model of Alzheimer's disease. Antioxidants (Basel) 10:1657. doi: 10.3390/antiox10111657, PMID: 34829528 PMC8614967

[ref76] Khalil M. Renner A. Langkammer C. Enzinger C. Ropele S. Stojakovic T.. (2016). Cerebrospinal fluid lipocalin 2 in patients with clinically isolated syndromes and early multiple sclerosis. Mult. Scler. 22, 1560–1568. doi: 10.1177/1352458515624560, PMID: 26762671

[ref77] Kiaei M. Kipiani K. Calingasan N. Y. Wille E. Chen J. Heissig B.. (2007). Matrix metalloproteinase-9 regulates TNF-alpha and FasL expression in neuronal, glial cells and its absence extends life in a transgenic mouse model of amyotrophic lateral sclerosis. Exp. Neurol. 205, 74–81. doi: 10.1016/j.expneurol.2007.01.036, PMID: 17362932

[ref78] Kiernan M. C. Vucic S. Cheah B. C. Turner M. R. Eisen A. Hardiman O.. (2011). Amyotrophic lateral sclerosis. Lancet 377, 942–955. doi: 10.1016/s0140-6736(10)61156-7, PMID: 21296405

[ref79] Kim J. H. Jeong H. G. Hyeon S. J. Park U. Oh W. J. Hwang J.. (2025). Crosstalk between lipocalin-2 and IL-6 in traumatic brain injury: closely related biomarkers. Exp. Neurol. 385:115092. doi: 10.1016/j.expneurol.2024.115092, PMID: 39637963

[ref80] Kim B. W. Jeong K. H. Kim J. H. Jin M. Kim J. H. Lee M. G.. (2016). Pathogenic upregulation of glial Lipocalin-2 in the parkinsonian dopaminergic system. J. Neurosci. 36, 5608–5622. doi: 10.1523/jneurosci.4261-15.2016, PMID: 27194339 PMC6601774

[ref81] Kim J. H. Kang R. J. Hyeon S. J. Ryu H. Joo H. Bu Y.. (2023). Lipocalin-2 is a key regulator of neuroinflammation in secondary traumatic and ischemic brain injury. Neurotherapeutics 20, 803–821. doi: 10.1007/s13311-022-01333-5, PMID: 36508119 PMC10275845

[ref82] Kjeldsen L. Bainton D. F. Sengeløv H. Borregaard N. (1994). Identification of neutrophil gelatinase-associated lipocalin as a novel matrix protein of specific granules in human neutrophils. Blood 83, 799–807. doi: 10.1182/blood.V83.3.799.799, PMID: 8298140

[ref83] Kjeldsen L. Johnsen A. H. Sengeløv H. Borregaard N. (1993). Isolation and primary structure of NGAL, a novel protein associated with human neutrophil gelatinase. J. Biol. Chem. 268, 10425–10432. doi: 10.1016/S0021-9258(18)82217-7, PMID: 7683678

[ref84] Klavžar P. Koritnik B. Leonardis L. Dolenc Grošelj L. Kirbiš M. Ristić Kovačič S.. (2020). Improvements in the multidisciplinary care are beneficial for survival in amyotrophic lateral sclerosis (ALS): experience from a tertiary ALS center. Amyotroph. Lateral Scler. Frontotemporal Degener. 21, 203–208. doi: 10.1080/21678421.2020.1746809, PMID: 32248716

[ref85] Kubben F. J. Sier C. F. Hawinkels L. J. Tschesche H. van Duijn W. Zuidwijk K.. (2007). Clinical evidence for a protective role of lipocalin-2 against MMP-9 autodegradation and the impact for gastric cancer. Eur. J. Cancer 43, 1869–1876. doi: 10.1016/j.ejca.2007.05.013, PMID: 17604154

[ref86] Langelueddecke C. Lee W. K. Thévenod F. (2014). Differential transcytosis and toxicity of the hNGAL receptor ligands cadmium-metallothionein and cadmium-phytochelatin in colon-like Caco-2 cells: implications for in vivo cadmium toxicity. Toxicol. Lett. 226, 228–235. doi: 10.1016/j.toxlet.2014.01.049, PMID: 24518829

[ref87] Langelueddecke C. Roussa E. Fenton R. A. Wolff N. A. Lee W. K. Thévenod F. (2012). Lipocalin-2 (24p3/neutrophil gelatinase-associated lipocalin (NGAL)) receptor is expressed in distal nephron and mediates protein endocytosis. J. Biol. Chem. 287, 159–169. doi: 10.1074/jbc.M111.308296, PMID: 22084236 PMC3249067

[ref88] Laohavisudhi K. Sriwichaiin S. Attachaipanich T. Wittayachamnankul B. Chattipakorn N. Chattipakorn S. (2024). Mechanistic insights into Lipocalin-2 in ischemic stroke and hemorrhagic brain injury: integrating animal and clinical studies. Exp. Neurol. 379:114885. doi: 10.1016/j.expneurol.2024.114885, PMID: 38996863

[ref89] Larsen M. T. Häger M. Glenthøj A. Asmar F. Clemmensen S. N. Mora-Jensen H.. (2014). miRNA-130a regulates C/EBP-ε expression during granulopoiesis. Blood 123, 1079–1089. doi: 10.1182/blood-2013-08-523233, PMID: 24398327

[ref90] Lee S. Lee J. Kim S. Park J. Y. Lee W. H. Mori K.. (2007). A dual role of lipocalin 2 in the apoptosis and deramification of activated microglia. J. Immunol. 179, 3231–3241. doi: 10.4049/jimmunol.179.5.3231, PMID: 17709539

[ref91] Lee S. Lee W. H. Lee M. S. Mori K. Suk K. (2012). Regulation by lipocalin-2 of neuronal cell death, migration, and morphology. J. Neurosci. Res. 90, 540–550. doi: 10.1002/jnr.22779, PMID: 22038922

[ref92] Lee S. Park J. Y. Lee W. H. Kim H. Park H. C. Mori K.. (2009). Lipocalin-2 is an autocrine mediator of reactive astrocytosis. J. Neurosci. 29, 234–249. doi: 10.1523/jneurosci.5273-08.2009, PMID: 19129400 PMC6664907

[ref93] Li X. Wang X. Guo L. Wu K. Wang L. Rao L.. (2023). Association between lipocalin-2 and mild cognitive impairment or dementia: a systematic review and meta-analysis of population-based evidence. Ageing Res. Rev. 89:101984. doi: 10.1016/j.arr.2023.101984, PMID: 37330019

[ref94] Li J. Xu P. Hong Y. Xie Y. Peng M. Sun R.. (2023). Lipocalin-2-mediated astrocyte pyroptosis promotes neuroinflammatory injury via NLRP3 inflammasome activation in cerebral ischemia/reperfusion injury. J. Neuroinflammation 20:148. doi: 10.1186/s12974-023-02819-5, PMID: 37353794 PMC10288712

[ref95] Liddelow S. A. Barres B. A. (2017). Reactive astrocytes: production, function, and therapeutic potential. Immunity 46, 957–967. doi: 10.1016/j.immuni.2017.06.006 28636962

[ref96] Liddelow S. A. Guttenplan K. A. Clarke L. E. Bennett F. C. Bohlen C. J. Schirmer L.. (2017). Neurotoxic reactive astrocytes are induced by activated microglia. Nature 541, 481–487. doi: 10.1038/nature21029, PMID: 28099414 PMC5404890

[ref97] Lim D. Jeong J. H. Song J. (2021). Lipocalin 2 regulates iron homeostasis, neuroinflammation, and insulin resistance in the brains of patients with dementia: evidence from the current literature. CNS Neurosci. Ther. 27, 883–894. doi: 10.1111/cns.13653, PMID: 33945675 PMC8265939

[ref98] Lim L. Song J. (2015). A novel SOD1-dependent mechanism for the iron-induced production of toxic SOD1 and oxidative stress that initiates ALS. bioRxiv:018846. doi: 10.1101/018846 [Preprint].

[ref99] Liu E. Karpf L. Bohl D. (2021). Neuroinflammation in amyotrophic lateral sclerosis and frontotemporal dementia and the interest of induced pluripotent stem cells to study immune cells interactions with neurons. Front. Mol. Neurosci. 14:767041. doi: 10.3389/fnmol.2021.767041, PMID: 34970118 PMC8712677

[ref100] Liu R. Wang J. Chen Y. Collier J. M. Capuk O. Jin S.. (2022). NOX activation in reactive astrocytes regulates astrocytic LCN2 expression and neurodegeneration. Cell Death Dis. 13:371. doi: 10.1038/s41419-022-04831-8, PMID: 35440572 PMC9018876

[ref101] Liu Y. Yao Q. Yu J. Zhang Y. Xiao Y. Zhang N.. (2025). The bone-heart axis: a crucial dialogue in cardiovascular disease. Metabolism 170:156332. doi: 10.1016/j.metabol.2025.156332, PMID: 40543811

[ref102] Loane D. J. Byrnes K. R. (2010). Role of microglia in neurotrauma. Neurotherapeutics 7, 366–377. doi: 10.1016/j.nurt.2010.07.002, PMID: 20880501 PMC2948548

[ref103] Longhurst J. C. Tjen A. L. S. C. (2017). Evidence-based blood pressure reducing actions of electroacupuncture: mechanisms and clinical application. Sheng Li Xue Bao 69, 587–597, PMID: 29063107 PMC6033058

[ref104] Marin B. Boumédiene F. Logroscino G. Couratier P. Babron M. C. Leutenegger A. L.. (2017). Variation in worldwide incidence of amyotrophic lateral sclerosis: a meta-analysis. Int. J. Epidemiol. 46, dyw061–dyw074. doi: 10.1093/ije/dyw061, PMID: 27185810 PMC5407171

[ref105] Marin B. Fontana A. Arcuti S. Copetti M. Boumédiene F. Couratier P.. (2018). Age-specific ALS incidence: a dose-response meta-analysis. Eur. J. Epidemiol. 33, 621–634. doi: 10.1007/s10654-018-0392-x, PMID: 29687175

[ref106] Marzolo M. P. Farfán P. (2011). New insights into the roles of megalin/LRP2 and the regulation of its functional expression. Biol. Res. 44, 89–105. doi: 10.4067/s0716-97602011000100012, PMID: 21720686

[ref107] McWilliam S. J. Antoine D. J. Sabbisetti V. Pearce R. E. Jorgensen A. L. Lin Y.. (2014). Reference intervals for urinary renal injury biomarkers KIM-1 and NGAL in healthy children. Biomark. Med 8, 1189–1197. doi: 10.2217/bmm.14.36, PMID: 24661102 PMC4076175

[ref108] Mehta P. Kaye W. Raymond J. Punjani R. Larson T. Cohen J.. (2018). Prevalence of amyotrophic lateral sclerosis - United States, 2015. MMWR Morb. Mortal Wkly. Rep. 67, 1285–1289. doi: 10.15585/mmwr.mm6746a1, PMID: 30462626 PMC6289079

[ref109] Menon P. Kiernan M. C. Vucic S. (2015). Cortical hyperexcitability precedes lower motor neuron dysfunction in ALS. Clin. Neurophysiol. 126, 803–809. doi: 10.1016/j.clinph.2014.04.023, PMID: 25227219

[ref110] Mesquita S. D. Ferreira A. C. Falcao A. M. Sousa J. C. Oliveira T. G. Correia-Neves M.. (2014). Lipocalin 2 modulates the cellular response to amyloid beta. Cell Death Differ. 21, 1588–1599. doi: 10.1038/cdd.2014.68, PMID: 24853299 PMC4158684

[ref111] Mollinedo F. (2019). Neutrophil degranulation, plasticity, and Cancer metastasis. Trends Immunol. 40, 228–242. doi: 10.1016/j.it.2019.01.006, PMID: 30777721

[ref112] Moreau C. Danel V. Devedjian J. C. Grolez G. Timmerman K. Laloux C.. (2018). Could conservative Iron chelation Lead to neuroprotection in amyotrophic lateral sclerosis? Antioxid. Redox Signal. 29, 742–748. doi: 10.1089/ars.2017.7493, PMID: 29287521 PMC6067092

[ref113] Mosialou I. Shikhel S. Liu J. M. Maurizi A. Luo N. He Z.. (2017). MC4R-dependent suppression of appetite by bone-derived lipocalin 2. Nature 543, 385–390. doi: 10.1038/nature21697, PMID: 28273060 PMC5975642

[ref114] Murdock B. J. Goutman S. A. Boss J. Kim S. Feldman E. L. (2021). Amyotrophic lateral sclerosis survival associates with neutrophils in a sex-specific manner. Neurol. Neuroimmunol. Neuroinflamm. 8:e953. doi: 10.1212/nxi.0000000000000953, PMID: 33531377 PMC8057067

[ref115] Murdock B. J. Zhou T. Kashlan S. R. Little R. J. Goutman S. A. Feldman E. L. (2017). Correlation of peripheral immunity with rapid amyotrophic lateral sclerosis progression. JAMA Neurol. 74, 1446–1454. doi: 10.1001/jamaneurol.2017.2255, PMID: 28973548 PMC5822195

[ref116] Nairz M. Schroll A. Haschka D. Dichtl S. Sonnweber T. Theurl I.. (2015). Lipocalin-2 ensures host defense against *Salmonella Typhimurium* by controlling macrophage iron homeostasis and immune response. Eur. J. Immunol. 45, 3073–3086. doi: 10.1002/eji.201545569, PMID: 26332507 PMC4688458

[ref117] Nairz M. Theurl I. Schroll A. Theurl M. Fritsche G. Lindner E.. (2009). Absence of functional Hfe protects mice from invasive *Salmonella enterica* serovar typhimurium infection via induction of lipocalin-2. Blood 114, 3642–3651. doi: 10.1182/blood-2009-05-223354, PMID: 19700664 PMC2766679

[ref118] Nakagawa Y. Chiba K. (2015). Diversity and plasticity of microglial cells in psychiatric and neurological disorders. Pharmacol. Ther. 154, 21–35. doi: 10.1016/j.pharmthera.2015.06.010, PMID: 26129625

[ref119] Nardo G. Iennaco R. Fusi N. Heath P. R. Marino M. Trolese M. C.. (2013). Transcriptomic indices of fast and slow disease progression in two mouse models of amyotrophic lateral sclerosis. Brain 136, 3305–3332. doi: 10.1093/brain/awt250, PMID: 24065725

[ref120] Naudé P. J. Nyakas C. Eiden L. E. Ait-Ali D. van der Heide R. Engelborghs S.. (2012). Lipocalin 2: novel component of proinflammatory signaling in Alzheimer's disease. FASEB J. 26, 2811–2823. doi: 10.1096/fj.11-202457, PMID: 22441986 PMC3382095

[ref121] Ngo S. T. Steyn F. J. Huang L. Mantovani S. Pfluger C. M. Woodruff T. M.. (2015). Altered expression of metabolic proteins and adipokines in patients with amyotrophic lateral sclerosis. J. Neurol. Sci. 357, 22–27. doi: 10.1016/j.jns.2015.06.053, PMID: 26198021

[ref122] Noçon A. L. Ip J. P. Terry R. Lim S. L. Getts D. R. Müller M.. (2014). The bacteriostatic protein lipocalin 2 is induced in the central nervous system of mice with West Nile virus encephalitis. J. Virol. 88, 679–689. doi: 10.1128/jvi.02094-13, PMID: 24173226 PMC3911733

[ref123] O'Neill K. Shaw R. Bolger I. Tam O. H. Phatnani H. Gale Hammell M. (2025). ALS molecular subtypes are a combination of cellular and pathological features learned by deep multiomics classifiers. Cell Rep. 44:115402. doi: 10.1016/j.celrep.2025.115402, PMID: 40067829 PMC12011103

[ref124] Petillon C. Hergesheimer R. Puy H. Corcia P. Vourc'h P. Andres C.. (2018). The relevancy of data regarding the metabolism of Iron to our understanding of deregulated mechanisms in ALS; Hypotheses and Pitfalls. Front. Neurosci. 12:1031. doi: 10.3389/fnins.2018.01031, PMID: 30697143 PMC6341213

[ref125] Petrozziello T. Mills A. N. Farhan S. M. K. Mueller K. A. Granucci E. J. Glajch K. E.. (2020). Lipocalin-2 is increased in amyotrophic lateral sclerosis. Muscle Nerve 62, 272–283. doi: 10.1002/mus.26911, PMID: 32369618

[ref126] Richardson D. R. (2005). 24p3 and its receptor: dawn of a new iron age? Cell 123, 1175–1177. doi: 10.1016/j.cell.2005.12.008, PMID: 16377555

[ref127] Schröder S. K. Gasterich N. Weiskirchen S. Weiskirchen R. (2023). Lipocalin 2 receptors: facts, fictions, and myths. Front. Immunol. 14:1229885. doi: 10.3389/fimmu.2023.1229885, PMID: 37638032 PMC10451079

[ref128] Shibata N. Yamamoto T. Hiroi A. Omi Y. Kato Y. Kobayashi M. (2010). Activation of STAT3 and inhibitory effects of pioglitazone on STAT3 activity in a mouse model of SOD1-mutated amyotrophic lateral sclerosis. Neuropathology 30, 353–360. doi: 10.1111/j.1440-1789.2009.01078.x, PMID: 19925559

[ref129] Shiratori-Hayashi M. Yamaguchi C. Eguchi K. Shiraishi Y. Kohno K. Mikoshiba K.. (2021). Astrocytic STAT3 activation and chronic itch require IP(3)R1/TRPC-dependent ca(2+) signals in mice. J. Allergy Clin. Immunol. 147, 1341–1353. doi: 10.1016/j.jaci.2020.06.039, PMID: 32781002

[ref130] Somjen G. G. (1988). Nervenkitt: notes on the history of the concept of neuroglia. Glia 1, 2–9. doi: 10.1002/glia.440010103, PMID: 2976736

[ref131] Song E. Fan P. Huang B. Deng H. B. Cheung B. M. Félétou M.. (2014). Deamidated lipocalin-2 induces endothelial dysfunction and hypertension in dietary obese mice. J. Am. Heart Assoc. 3:e000837. doi: 10.1161/jaha.114.000837, PMID: 24721803 PMC4187505

[ref132] Spiller K. J. Khan T. Dominique M. A. Restrepo C. R. Cotton-Samuel D. Levitan M.. (2019). Reduction of matrix metalloproteinase 9 (MMP-9) protects motor neurons from TDP-43-triggered death in rNLS8 mice. Neurobiol. Dis. 124, 133–140. doi: 10.1016/j.nbd.2018.11.013, PMID: 30458231 PMC7053168

[ref133] Suk K. (2016). Lipocalin-2 as a therapeutic target for brain injury: An astrocentric perspective. Prog. Neurobiol. 144, 158–172. doi: 10.1016/j.pneurobio.2016.08.001, PMID: 27498195

[ref134] Sung C. Y. Chiang P. K. Tsai C. W. Yang F. Y. (2021). Low-intensity pulsed ultrasound enhances neurotrophic factors and alleviates neuroinflammation in a rat model of Parkinson's disease. Cereb. Cortex 32, 176–185. doi: 10.1093/cercor/bhab201, PMID: 34196669

[ref135] Tan Q. Zhang C. Rao X. Wan W. Lin W. Huang S.. (2024). The interaction of lipocalin-2 and astrocytes in neuroinflammation: mechanisms and therapeutic application. Front. Immunol. 15:1358719. doi: 10.3389/fimmu.2024.1358719, PMID: 38533497 PMC10963420

[ref136] Tao Y. X. (2010). The melanocortin-4 receptor: physiology, pharmacology, and pathophysiology. Endocr. Rev. 31, 506–543. doi: 10.1210/er.2009-0037, PMID: 20190196 PMC3365848

[ref137] Thal D. R. Gawor K. Moonen S. (2024). Regulated cell death and its role in Alzheimer's disease and amyotrophic lateral sclerosis. Acta Neuropathol. 147:69. doi: 10.1007/s00401-024-02722-0, PMID: 38583129

[ref138] Tong J. Huang C. Bi F. Wu Q. Huang B. Liu X.. (2013). Expression of ALS-linked TDP-43 mutant in astrocytes causes non-cell-autonomous motor neuron death in rats. EMBO J. 32, 1917–1926. doi: 10.1038/emboj.2013.122, PMID: 23714777 PMC3981181

[ref139] Tschesche H. Zölzer V. Triebel S. Bartsch S. (2001). The human neutrophil lipocalin supports the allosteric activation of matrix metalloproteinases. Eur. J. Biochem. 268, 1918–1928. doi: 10.1046/j.1432-1327.2001.02066.x, PMID: 11277914

[ref140] Tuddenham J. F. Fujita M. Lee E. Nimmagadda N. Khairallah A. Harbison C.. (2025). Single-cell transcriptomic landscape of the neuroimmune compartment in amyotrophic lateral sclerosis brain and spinal cord. Acta Neuropathol. 150:10. doi: 10.1007/s00401-025-02913-3, PMID: 40728732 PMC12307561

[ref141] Van den Steen P. E. Van Aelst I. Hvidberg V. Piccard H. Fiten P. Jacobsen C.. (2006). The hemopexin and O-glycosylated domains tune gelatinase B/MMP-9 bioavailability via inhibition and binding to cargo receptors. J. Biol. Chem. 281, 18626–18637. doi: 10.1074/jbc.M512308200, PMID: 16672230

[ref142] Verma S. Khurana S. Vats A. Sahu B. Ganguly N. K. Chakraborti P.. (2022). Neuromuscular junction dysfunction in amyotrophic lateral sclerosis. Mol. Neurobiol. 59, 1502–1527. doi: 10.1007/s12035-021-02658-6 34997540

[ref143] Vismara I. Papa S. Veneruso V. Mauri E. Mariani A. De Paola M.. (2020). Selective modulation of A1 astrocytes by drug-loaded Nano-structured gel in spinal cord injury. ACS Nano 14, 360–371. doi: 10.1021/acsnano.9b05579, PMID: 31887011

[ref144] Wang Z. Cao W. Chen L. Zhang S. Tang L. Cui W.. (2025). The role of the peripheral immune system in mediating axonal dysfunction in early-stage amyotrophic lateral sclerosis: an age- and sex-based analysis. Neural Regen. Res. doi: 10.4103/nrr.Nrr-d-24-01081 [a head of print]., PMID: 40145976 PMC13378947

[ref145] Wang Y. Lam K. S. Kraegen E. W. Sweeney G. Zhang J. Tso A. W.. (2007). Lipocalin-2 is an inflammatory marker closely associated with obesity, insulin resistance, and hyperglycemia in humans. Clin. Chem. 53, 34–41. doi: 10.1373/clinchem.2006.075614, PMID: 17040956

[ref146] Wang L. Li H. Wang J. Gao W. Lin Y. Jin W.. (2011). C/EBP ζ targets to neutrophil gelatinase-associated lipocalin (NGAL) as a repressor for metastasis of MDA-MB-231 cells. Biochim. Biophys. Acta 1813, 1803–1813. doi: 10.1016/j.bbamcr.2011.06.010, PMID: 21741997

[ref147] Wang X. Li X. Zuo X. Liang Z. Ding T. Li K.. (2021). Photobiomodulation inhibits the activation of neurotoxic microglia and astrocytes by inhibiting Lcn2/JAK2-STAT3 crosstalk after spinal cord injury in male rats. J. Neuroinflammation 18:256. doi: 10.1186/s12974-021-02312-x, PMID: 34740378 PMC8571847

[ref148] Wang B. Wang L. N. Wu B. Guo R. Zhang L. Zhang J. T.. (2024). Astrocyte PERK and IRE1 Signaling contributes to morphine tolerance and hyperalgesia through upregulation of Lipocalin-2 and NLRP3 inflammasome in the rodent spinal cord. Anesthesiology 140, 558–577. doi: 10.1097/aln.0000000000004858, PMID: 38079113

[ref149] Wang G. Weng Y. C. Chiang I. C. Huang Y. T. Liao Y. C. Chen Y. C.. (2020). Neutralization of Lipocalin-2 diminishes stroke-reperfusion injury. Int. J. Mol. Sci. 21:6253. doi: 10.3390/ijms21176253, PMID: 32872405 PMC7503651

[ref150] Wang Q. Zhang X. Chen S. Zhang X. Zhang S. Youdium M.. (2011). Prevention of motor neuron degeneration by novel iron chelators in SOD1(G93A) transgenic mice of amyotrophic lateral sclerosis. Neurodegener Dis 8, 310–321. doi: 10.1159/000323469, PMID: 21346313

[ref151] Ward R. J. Zucca F. A. Duyn J. H. Crichton R. R. Zecca L. (2014). The role of iron in brain ageing and neurodegenerative disorders. Lancet Neurol. 13, 1045–1060. doi: 10.1016/s1474-4422(14)70117-6, PMID: 25231526 PMC5672917

[ref152] Wei L. Du Y. Xie Y. Yu X. Chen H. Qiu Y. (2021). Lipocalin-2 regulates hippocampal microglial activation in poststroke depression. Front. Aging Neurosci. 13:798335. doi: 10.3389/fnagi.2021.798335, PMID: 34966272 PMC8710735

[ref153] Willman M. Patel G. Lucke-Wold B. (2024). T lymphocyte proportion in Alzheimer's disease prognosis. World J. Clin. Cases 12, 6001–6003. doi: 10.12998/wjcc.v12.i26.6001, PMID: 39286389 PMC11287512

[ref154] Wyss-Coray T. Mucke L. (2002). Inflammation in neurodegenerative disease--a double-edged sword. Neuron 35, 419–432. doi: 10.1016/s0896-6273(02)00794-8 12165466

[ref155] Xia K. Zhang L. Tang L. Huang T. Fan D. (2022). Assessing the role of blood pressure in amyotrophic lateral sclerosis: a mendelian randomization study. Orphanet J. Rare Dis. 17:56. doi: 10.1186/s13023-022-02212-0, PMID: 35172853 PMC8848798

[ref156] Xiang X. Tang X. Yu Y. Xie S. Liu L. Chen M.. (2022). Role of lipocalin-2 in surgery-induced cognitive decline in mice: a signal from neuron to microglia. J. Neuroinflammation 19:92. doi: 10.1186/s12974-022-02455-5, PMID: 35413913 PMC9006597

[ref157] Xiao R. Pan J. Yang M. Liu H. Zhang A. Guo X.. (2025). Regulating astrocyte phenotype by Lcn2 inhibition toward ischemic stroke therapy. Biomaterials 317:123102. doi: 10.1016/j.biomaterials.2025.123102, PMID: 39836995

[ref158] Xiao X. Yeoh B. S. Vijay-Kumar M. (2017). Lipocalin 2: An emerging player in Iron homeostasis and inflammation. Annu. Rev. Nutr. 37, 103–130. doi: 10.1146/annurev-nutr-071816-064559, PMID: 28628361

[ref159] Xie Y. Zhuo X. Xing K. Huang Z. Guo H. Gong P.. (2023). Circulating lipocalin-2 as a novel biomarker for early neurological deterioration and unfavorable prognosis after acute ischemic stroke. Brain Behav. 13:e2979. doi: 10.1002/brb3.2979, PMID: 36974345 PMC10176013

[ref160] Xiong H. Luo T. He W. Xi D. Lu H. Li M.. (2016). Up-regulation of miR-138 inhibits hypoxia-induced cardiomyocyte apoptosis via down-regulating lipocalin-2 expression. Exp. Biol. Med. (Maywood) 241, 25–30. doi: 10.1177/1535370215591831, PMID: 26129883 PMC4935435

[ref161] Xiong M. Qian Q. Liang X. Wei Y. D. (2022). Serum levels of lipocalin-2 in patients with Parkinson's disease. Neurol. Sci. 43, 1755–1759. doi: 10.1007/s10072-021-05579-3, PMID: 34455500

[ref162] Yamaguchi N. Sawano T. Nakatani J. Nakano-Doi A. Nakagomi T. Matsuyama T.. (2023). Voluntary running exercise modifies astrocytic population and features in the peri-infarct cortex. IBRO Neurosci. Rep. 14, 253–263. doi: 10.1016/j.ibneur.2023.02.004, PMID: 36880055 PMC9984846

[ref163] Yazdani S. Seitz C. Cui C. Lovik A. Pan L. Piehl F.. (2022). T cell responses at diagnosis of amyotrophic lateral sclerosis predict disease progression. Nat. Commun. 13:6733. doi: 10.1038/s41467-022-34526-9, PMID: 36347843 PMC9643478

[ref164] Yndestad A. Landrø L. Ueland T. Dahl C. P. Flo T. H. Vinge L. E.. (2009). Increased systemic and myocardial expression of neutrophil gelatinase-associated lipocalin in clinical and experimental heart failure. Eur. Heart J. 30, 1229–1236. doi: 10.1093/eurheartj/ehp088, PMID: 19329498

[ref165] Yu H. Chen L. Zhang S. He J. Fan D. (2021). Early axonal dysfunction of the peripheral nervous system influences disease progression of ALS: evidence from clinical Neuroelectrophysiology. Front. Neurol. 12:574919. doi: 10.3389/fneur.2021.574919, PMID: 33643181 PMC7905229

[ref166] Yu W. He J. Cai X. Yu Z. Zou Z. Fan D. (2022). Neuroimmune crosstalk between the peripheral and the central immune system in amyotrophic lateral sclerosis. Front. Aging Neurosci. 14:890958. doi: 10.3389/fnagi.2022.890958, PMID: 35592701 PMC9110796

[ref167] Zejlon C. Sennfält S. Finnsson J. Connolly B. Petersson S. Granberg T.. (2024). Motor band sign is specific for amyotrophic lateral sclerosis and corresponds to motor symptoms. Ann. Clin. Transl. Neurol. 11, 1280–1289. doi: 10.1002/acn3.52066, PMID: 38647181 PMC11093233

[ref168] Zelic M. Blazier A. Pontarelli F. LaMorte M. Huang J. Tasdemir-Yilmaz O. E.. (2025). Single-cell transcriptomic and functional studies identify glial state changes and a role for inflammatory RIPK1 signaling in ALS pathogenesis. Immunity 58, 961–979.e8. doi: 10.1016/j.immuni.2025.02.024, PMID: 40132594

[ref169] Zhang N. Aiyasiding X. Li W. J. Liao H. H. Tang Q. Z. (2022). Neutrophil degranulation and myocardial infarction. Cell Commun. Signal 20:50. doi: 10.1186/s12964-022-00824-4, PMID: 35410418 PMC8996539

[ref170] Zhang Y. Liu J. Yao M. Song W. Zheng Y. Xu L.. (2019). Sailuotong capsule prevents the cerebral ischaemia-induced neuroinflammation and impairment of recognition memory through inhibition of LCN2 expression. Oxidative Med. Cell. Longev. 2019, 1–13. doi: 10.1155/2019/8416105, PMID: 31565154 PMC6745154

[ref171] Zhang Q. N. Yu L. K. Zhang X. Y. Wu Y. Zhang H. Wu J. L.. (2025). LCN2 of cerebrospinal fluid: a potential biomarker for diagnosis and disease progression in Alzheimer's disease. J Alzheimer's Dis 106, 1573–1581. doi: 10.1177/13872877251352411, PMID: 40576449

[ref172] Zhang P. X. Zhang F. R. Xie J. J. Tao L. H. Lü Z. Xu X. E.. (2012). Expression of NGAL and NGALR in human embryonic, fetal and normal adult tissues. Mol. Med. Rep. 6, 716–722. doi: 10.3892/mmr.2012.980, PMID: 22797813

[ref173] Zhao R. Y. Wei P. J. Sun X. Zhang D. H. He Q. Y. Liu J.. (2023). Role of lipocalin 2 in stroke. Neurobiol. Dis. 179:106044. doi: 10.1016/j.nbd.2023.106044, PMID: 36804285

[ref174] Zhao N. Xu X. Jiang Y. Gao J. Wang F. Xu X.. (2019). Lipocalin-2 may produce damaging effect after cerebral ischemia by inducing astrocytes classical activation. J. Neuroinflammation 16:168. doi: 10.1186/s12974-019-1556-7, PMID: 31426811 PMC6699078

